# Sigma-2 receptor ligands QSAR model dataset

**DOI:** 10.1016/j.dib.2017.06.022

**Published:** 2017-06-16

**Authors:** Antonio Rescifina, Giuseppe Floresta, Agostino Marrazzo, Carmela Parenti, Orazio Prezzavento, Giovanni Nastasi, Maria Dichiara, Emanuele Amata

**Affiliations:** a Department of Drug Sciences, University of Catania, Viale A. Doria 6, 95125 Catania, Italy; b Department of Chemical Sciences, University of Catania, Viale A. Doria 6, 95125 Catania, Italy; c Department of Mathematics and Computer Sciences, University of Catania, Viale A. Doria 6, 95125 Catania, Italy

## Abstract

The data have been obtained from the Sigma-2 Receptor Selective Ligands Database (S2RSLDB) and refined according to the QSAR requirements. These data provide information about a set of 548 Sigma-2 (σ_2_) receptor ligands selective over Sigma-1 (σ_1_) receptor. The development of the QSAR model has been undertaken with the use of CORAL software using SMILES, molecular graphs and hybrid descriptors (SMILES and graph together). Data here reported include the regression for σ_2_ receptor p*K*_i_ QSAR models. The QSAR model was also employed to predict the σ_2_ receptor p*K*_i_ values of the FDA approved drugs that are herewith included.

**Specifications Table**

**Table:** 

Subject area	Computational Chemistry
More specific subject area	Quantitative Structure-Activity Relationship (QSAR) modeling
Type of data	Table, figure
How data was acquired	Statistical modeling and online databases
Data format	Raw and analyzed
Experimental factors	The whole dataset consists of 548 σ_2_ receptor selective ligands which were randomly split and divided into training, invisible training, calibration, and validation sets.
Experimental features	The QSAR models have been developed using CORAL software. Chemical structure descriptors and p*K*_i_ were used as variables.
Data source location	Department of Drug Sciences, Department of Chemical Sciences, Department of Mathematics and Computer Sciences, University of Catania, Italy
Data accessibility	With this article

**Value of the data**

• The σ_2_ receptor is an important target overexpressed in several tumor cell lines and its ligands are currently under clinical evaluation as radiotracers and fluorescence agents.
• QSAR modeling data was generated to provide a method useful in finding or repurposing novel σ_2_ receptor ligands.
• The model has also been used to predict the σ_2_ receptor p*K*_i_ for the FDA-approved drugs.

## 1 Data

The sigma-2 (σ_2_) receptor is a peculiar target overexpressed in several tumor cell lines and its ligands are actually under clinical evaluation as positron emission tomography radiotracer and as fluorescence imaging agents [1], [2], [3]. Few selective ligands have been found for the σ_2_ receptor and in some cases, their finding occurred through an accidental discovery [1], [4]. Data here reported provide information about a set of σ_2_ receptor ligands, taken from the Sigma-2 Receptor Selective Ligands Database (S2RSLDB), and selective over σ_1_ receptor, together with their p*K*_i_ (–log*K*_i_) [5]. These latter have been used in building up the first hybrid QSAR model embracing the all set of known σ_2_ receptor selective ligands [6]. The model has also been used to predict the σ_2_ receptor p*K*_i_ for the Food and Drug Administration approved drugs. These latter predicted σ_2_ receptor p*K*_i_ data are also here reported.

## 2 Experimental design, materials and methods

### 2.1 Dataset preparation

The dataset consists of 548 σ_2_ receptor selective ligands which were randomly split three times and then divided into training (209 compounds), invisible training (209 compounds), calibration (65 compounds) sets for model development and a validation set (65 compounds) for invisible model validation. The three splits and four sets have been randomly generated, and their p*K*_i_ minimum, maximum and middle are reported in Table 1.

**Table 1:** Analysis of biological endpoint for hybrid models split 1 (p*K*_i_).

**Split**	**Set**	**Min**	**Max**	**Middle**
Split 1	Sub-training	5.49	9.68	7.58
	Calibration	5.28	11.21	7.49
	Test	5.75	10.27	7.74
	Validation	5.11	10.39	7.61
Split 2	Sub-training	5.15	10.39	7.56
	Calibration	5.28	11.21	7.48
	Test	5.44	9.48	7.72
	Validation	5.11	9.64	7.73
Split 3	Sub-training	5.49	10.39	7.58
	Calibration	5.11	11.21	7.57
	Test	5.87	9.25	7.46
	Validation	5.75	9.55	7.63

### 2.2 QSAR model development

QSAR models have been developed with the use of the software CORAL [7]. Once the splits and sets were determined, nine models were developed and statistical quality recorded. Differences of these models consist in the way molecular structures have been depicted the software. Thus, in Table 2 regressions for the σ_2_ receptor p*K*_i_ models using SMILES, molecular graphs and hybrid descriptors (SMILES and graph together) are reported. While in Table 3 is reported the statistical quality of models of the σ_2_ receptor p*K*_i_.

**Table 2:** Regression for the σ_2_ receptor p*K*_i_ models with CORAL.

**Model**	**Split**	**Regression equation**
**Hybrid**	Split 1	pKi_σ2=3.5937472(±0.0139734)+0.0352642(±0.0001213)*DCW(0,16)
	Split 2	pKi_σ2=-0.0004350(±0.0186857)+0.0669362(±0.0001660)*DCW(1,28)
	Split 3	pKi_σ2=2.3676460(±0.0172891)+0.0412001(±0.0001328) *DCW(0,18)
**SMILES**	Split 1	pKi_σ2=5.7680429(±0.0082114)+0.0679366(±0.0002918)*DCW(1,15)
	Split 2	pKi_σ2=5.2628160(±0.0099547)+0.0772202(±0.0003166)*DCW(1,15)
	Split 3	pKi_σ2=5.6659516(±0.0085196)+0.0672643(±0.0002911)*DCW(1,15)
**Graph**	Split 1	pKi_σ2=4.8227545(±0.0123932)+0.0523695(±0.0002307)*DCW(0,18)
	Split 2	pKi_σ2=5.7198837(±0.0113859)+0.0328674(±0.0001962)*DCW(3,14)
	Split 3	pKi_σ2=5.2942263(±0.0123925)+0.0367420(±0.0001880)*DCW(3,16)

**Table 3:** Statistical quality of models of the σ_2_ receptor p*K*_i_.

**Model**	**Split**	**Set**	T^*^	N^*^	***n***	***r***^**2**^	***q***^**2**^	***s***	***F***_**calc**_	***F***_(0.05,1,n–2)_	***p*****-Value**
**Hybrid**	Split 1	Sub-training	0	16	209	0.6475	0.6409	0.502	380	253.70	0.041
		Calibration			209	0.6475	0.6399	0.547	380	253.70	0.041
		Test			65	0.7463	0.7253	0.440	185	252.30	0.058
		Validation			65	0.7990		0.444	254	252.30	0.049
	Split 2	Sub-training	1	29	209	0.7659	0.7617	0.435	677	253.70	0.031
		Calibration			209	0.7364	0.7317	0.464	578	253.70	0.033
		Test			65	0.7672	0.7522	0.497	208	252.30	0.055
		Validation			65	0.7559		0.478	195	252.30	0.057
	Split 3	Sub-training	0	18	209	0.7087	0.7028	0.473	503	253.70	0.036
		Calibration			209	0.7117	0.7068	0.560	511	253.70	0.035
		Test			65	0.7755	0.7595	0.345	218	252.30	0.054
		Validation			65	0.7607		0.393	200	252.30	0.056
**SMILES**	Split 1	Sub-training	1	15	209	0.5273	0.5187	0.582	231	253.70	0.052
		Calibration			209	0.5289	0.5185	0.622	232	253.70	0.052
		Test			65	0.5631	0.5308	0.578	81	252.30	0.088
		Validation			65	0.6842		0.555	136	252.30	0.068
	Split 2	Sub-training	1	15	209	0.5570	0.5489	0.598	260	253.70	0.050
		Calibration			209	0.5427	0.5330	0.619	246	253.70	0.051
		Test			65	0.5956	0.5569	0.618	93	252.30	0.082
		Validation			65	0.6946		0.496	143	252.30	0.066
	Split 3	Sub-training	0	17	209	0.5766	0.5681	0.570	282	253.70	0.047
		Calibration			209	0.5758	0.5678	0.666	281	253.70	0.048
		Test			65	0.4679	0.4351	0.516	55	252.30	0.107
		Validation			65	0.5858		0.526	89	252.30	0.084
**Graph**	Split 1	Sub-training	0	18	209	0.5058	0.4969	0.595	212	253.70	0.055
		Calibration			209	0.4963	0.4852	0.645	204	253.70	0.056
		Test			65	0.5919	0.5334	0.565	91	252.30	0.083
		Validation			65	0.5804		0.652	87	252.30	0.085
	Split 2	Sub-training	3	14	209	0.4194	0.4076	0.684	150	253.70	0.065
		Calibration			209	0.4289	0.4163	0.690	155	253.70	0.064
		Test			65	0.6698	0.6480	0.503	128	252.30	0.070
		Validation			65	0.5989		0.527	94	252.30	0.082
	Split 3	Sub-training	3	16	209	0.4966	0.4864	0.621	204	253.70	0.056
		Calibration			209	0.4957	0.4864	0.697	203	253.70	0.056
		Test			65	0.4198	0.3804	0.542	46	252.30	0.117
		Validation			65	0.4358		0.608	49	252.30	0.113

T^*^ and N^*^ are preferable values for the threshold and the number of epochs, respectively; n is the number of compounds in the set; r^2^ is the correlation coefficient; q^2^ is the cross-validated correlation coefficient; s is the root-mean-square error; F is the Fisher F ratio; F_(0.05,1,n–2)_ is the 0.05-quantile of the Fisher׳s distribution F_(1,n–2)_; *p*-value is the Fisher test׳s significance level.

### 2.3 QSAR model settings for the best model [hybrid model split 1]

**Fig. 1:**
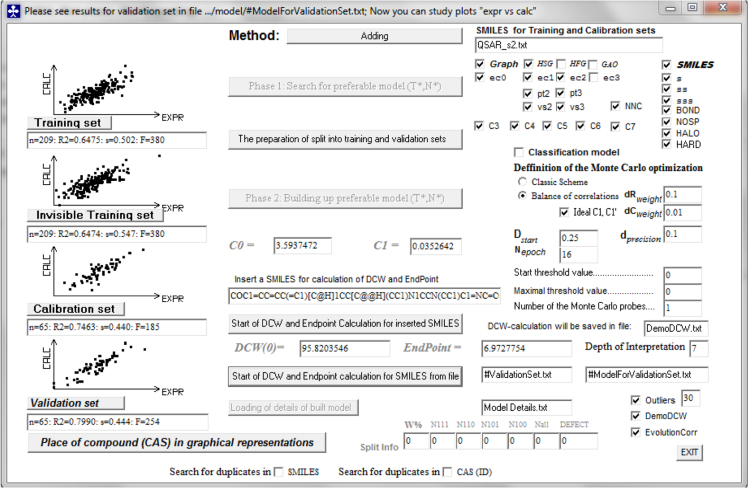
CORAL validation method for the σ_2_ receptor p*K*_i_ hybrid model Split 1.

Fig. 1 shows a CORAL screenshot with settings for hybrid model split 1. While in Table 4, the complete list of SMILES and their distribution into the sub-training (+), calibration (–), test (#) and validation (*) sets for σ_2_ receptor p*K*_i_ hybrid model split 1 is reported. These data may be prospectively used in finding novel models for σ_2_ receptor affinity.

**Table 4:** List of SMILES and their distribution into the sub-training (+), calibration (–), test (#) and validation (*) for hybrid model split 1.

–	549	[O-][N+](=O)C1=CC=C(C=C1)N1CCN(CCCCN2C(=O)OC3=CC=CC=C23)CC1	6.59176
–	548	COC1=C(NC(=O)OC2CC3CCCC(C2)N3CC2=CC=CC(F)=C2)C=C(C)C=C1	6.497573
–	547	O[C@@H]1CC[C@H](OCC2=CC=C(F)C=C2)C[C@H]1N(CC3)CCC3N4CCCCC4	6.2652
–	546	COC1=C(C=C(Br)C2=C1C=CC=C2)C(=O)NC[C@H]1CCCN1C1CCCCCC1	7.244125
–	542	CN(CC1CCCCC1)C1C2C3CC4C5CC(C2C35)C14	7.854
–	540	CN(CC1=CC=CN=C1)C1C2C3CC4C5CC(C2C35)C14	6.055024
–	539	C(CN1CC2=C(C1)C=CC=C2)CN1CCC2=C(C1)C1=CC=CC=C1O2	8.004365
–	538	COC1=C(C=C(Br)C2=C1C=CC=C2)C(=O)NC[C@H]1CCCN1CC1=CC=CC=C1	6.68403
–	534	FCCOCCOCCOC1=CC=C(C=C1)N1CCN(CCCCC2=CN(C3=C2C=CC=C3)C2=CC=C(F)C=C2)CC1	5.726073
–	533	OC(CCCN1CCC(O)(CC1)C1=CC=C(Cl)C=C1)C1=CC=C(F)C=C1	8.69897
–	529	COC1=C(C=C(Br)C2=C1C=CC=C2)C(=O)NC[C@H]1CCCN1C1C2CC3CC(C2)CC1C3	6.498941
–	528	BrC1=CC=C(NC(=O)OC2CC3CCCC(C2)N3CC2=CC=CC=C2)C=C1	7.962574
–	527	CC(COC1CC2CCC(C1)N2C)OC1=CC=C(Cl)C=C1	7
–	526	COC1=CC2=C(C=C1)C(CCCN1[C@@H](C)CCC[C@H]1C)CCC2	7.041436
–	517	COC1=C(OC)C(=CC(Br)=C1)C1=CC=C(CN2CC3=CC=CC=C3C2)N1	6.512862
–	509	COC1=CC=C(C=C1)[C@H]1CC[C@@H](CC1)N1CCN(CC1)C1=NC=CC=C1	6.653647
–	506	COC1=C(OC)C=C2CN(CCCCC(=O)C3=CC=C(F)C=C3)CCC2=C1	7.906578
–	505	FC1=CC=CC(F)=C1[C@H]1CC[C@@H](CC1)N1CCN(CC1)C1=NC=CC=C1	6.860121
–	503	COC1=C(NC(=O)OC2CC3CCCC(C2)N3CCCCCCNC(=O)C2=CC=C(I)C=C2)C=C(C)C=C1	5.996109
–	501	CN(CCN(C)CCN(C)CCC1=CC=C(Cl)C(Cl)=C1)CCN(C)CCN1CCCC1	6.790485
–	500	C[C@H]1CN(C[C@@H](C)N1CCCCN1C(=O)OC2=CC(=CC=C12)C(C)=O)C1=CC=C(F)C=C1	7.826814
–	499	CC(=O)N(CCCN1CCN(CC1)C1CCCCC1)C1=CC=CC=N1	6.609065
–	498	CC(OC1=CC=C(C=C1)C(C)(C)C)C(=O)OC1CC2CCC(C1)N2C	6.561
–	495	COC1=CC=C(NC2=CC=C3C[C@@H]4C5CCCC[C@]5(CCN4CC4CC4)C3=C2)C=C1	7.136677
–	494	CCCCCCCC1=CC=C2N(CCCCN3CCN(CC3)C3CCCCC3)C(=O)SC2=C1	7.533281
–	493	COC1=CC=CC2=C1CCCC2CCCN1CCN(CC1)C1CCN(CCCCCCNC2=CC=C(C3=NON=C23)[N+]([O-])=O)CC1	5.764472
–	492	C(NC1C2C3CC4C5CC(C2C35)C14)C12C3C4C5C3C1C5C24	8.699
–	486	O[C@@H]1CC2=C(C=CC=C2)C[C@H]1N(CC3)CCC3N4CCCCC4	6.793174
–	485	OC1(CCN(CCCC(OC(=O)CCCC2=CC=CC=C2)C2=CC=C(F)C=C2)CC1)C1=CC=C(Cl)C=C1	6.978811
–	482	CC1=CC=CC(C)=C1C1CCC(=CC1)N1CCN(CC1)C1=NC=CC=C1	6.947
–	479	COC1=CC(CNC2C3C4CC5C6CC(C3C46)C25)=CC(OC)=C1OC	6.202732
–	476	OC1(CCN(CCC[C@@H](OC(=O)CCCC2=CC=CC=C2)C2=CC=C(F)C=C2)CC1)C1=CC=C(Cl)C=C1	7.278189
–	472	COC1=CC(CN(C)C2C3C4CC5C6CC(C3C46)C25)=CC(OC)=C1OC	6.146302
–	469	COC1=CC2=C(CN(CC3CCN(CC3)C(=O)C3=CC=C(Br)C(C)=C3)CC2)C=C1OC	7.357
–	466	O=C(C1=CC=C(C=C1)F)NC2=C3C[C@H]([C@@H](CC3=CC=C2)O)N4CCC(CC4)C5=CC=CC=C5	5.441
–	464	CC1(C)CCCN(CCCC2=CC=CC3=C2C=C(O)C=C3)C1	6.385103
–	463	O=C1OC2=C(C=CC=C2)N1CCCCCN1CCN(CC1)C1CCCCC1	8.229885
–	462	CN(CC1=CC(F)=CC=C1)[C@@H]1C2C3C4C2C(=O)C2C4CC3C12	6.775
–	461	CCC(=O)C1=CC=C2N(CCN3CCN(CC3)C3CCCCC3)C(=O)SC2=C1	8.428291
–	458	COC1=CC=CC2=C1C=CC=C2NC(=O)CN1CCN(CC1)C1CCCCC1	7.635
–	453	COC1=C(OC)C2=C(C=C1)C(CC2)NCCCCC1=CN(C2=C1C=CC=C2)C1=CC=C(F)C=C1	6.939302
–	450	O=C(O[C@H](C1=CC=C(F)C=C1)CCCN2CCC(O)(C3=CC=C(Cl)C=C3)CC2)CCCC4=CC=CC=C4	6.928118
–	448	COC1=C(OC)C=C2C(C)N(CC3=CC=C(N3)C3=CC(Br)=CC(OC)=C3OC)CCC2=C1	6.30103
–	447	COC1=CC=CC2=C1CCC[C@H]2NC(=O)CN1CCN(CC1)C1CCCCC1	7.995679
–	446	COC1=CC=CC2=C1CCC[C@H]2NC(=O)CN1CCN(CC1)C1CCCCC1	7.995679
–	444	[H]OC1=CC=CC2=C1CCCC2CCCN1CCN(CC1)C1CCCCC1	8.575118
–	436	CC(C)C(SC1=CC=C(Cl)C=C1)C(=O)OC1CC2CCC(C1)N2C	6.479
–	432	COC1=C(OC)C2=C(C(N(CC)CCCCC3=CN(C4=CC=C(F)C=C4)C5=C3C=CC=C5)CC2)C=C1	7.559091
–	428	COC1=C(OC)C=C2CN(CC3=CC=C(N3)C3=CC(Br)=CC(OC)=C3OC)CCC2=C1	6.337242
–	425	COC1=C(C=C(Br)C2=C1C=CC=C2)C(=O)NC1CCN(CC1)C1C2CC3CC(C2)CC1C3	7.259637
–	419	FC1=CC=C(C=C1)N1C(=O)N(CCCCN2CCN(CC2)C2CCCCC2)C2=CC=CC=C12	8.673664
–	414	COC1=CC(CNCCCCC2=CN(C3=C2C=CC=C3)C2=CC=C(F)C=C2)=CC=C1	6.644
–	410	O=C1OC2=CC=CC=C2N1CCCCN1CCN(CC1)C1=CC=CC=N1	6.892451
–	409	[O-][N+](=O)C1=CC=C(NCCOCCOC2=CC=CC3=C2CCCC3CCCN2CCN(CC2)C2CCCCC2)C2=NON=C12	7.405607
–	408	COC1=CC2=C(CN(CC3CCN(CC3)C(=O)C3=CNC4=C3C=C(Br)C=C4)CC2)C=C1OC	6.79588
–	407	FC1=CC=C(C=C1)C1CCN(CCCCN2C3=CC=CC=C3C3=C2C=CC=C3)CC1	7.931814
–	402	C(CC1CCCC2=CC=CC=C12)CN1CCN(CC1)C1=NCCCC1	7.358526
–	399	COC1=CC=CC2=C1CCC[C@H]2NC(=O)CN1CCC2=CC(OC)=C(OC)C=C2C1	5.710857
–	398	CN(CC1=CC=CC=C1)[C@@H]1C2C3C4C2C(=O)C2C4CC3C12	6.664
–	396	COC1=C(OC)C2=C([C@@H](N(CC)CCCCC3=CN(C4=CC=C(F)C=C4)C5=C3C=CC=C5)CC2)C=C1	7.721
–	395	CCN(CCCCC1=CN(C2=C1C=CC=C2)C1=CC=C(F)C=C1)[C@H]1CCC2=C1C=CC(OC)=C2OC	7.721
–	390	COC1=CC=CC2=C1CCC[C@@H]2NCCN1CCN(CC1)C1CCCCC1	8.06956
–	386	COC1=CC=CC2=C1CCCC2NC(=O)CN1CCC2=CC(OC)=C(OC)C=C2C1	5.744727
–	385	COC1=CC=CC(=C1)[C@]12CCN(C)[C@H](C1)/C(=C/C1=CC=CC=C1)C(=O)C2	7.283997
–	384	COC1=C(O)C(=CC=C1)C(=O)NCCCCN1CCC2=CC=C(C=C2C1)[N+]([O-])=O	5.39794
–	382	CN(CCCC1=CC=CC=C1)[C@@H]1CCCC[C@H]1O	8.174
–	381	COC1=C(NC(=O)OC2CC3CCCC(C2)N3CCCCCCNC(=O)C2=CC=C(Br)C=C2)C=C(C)C=C1	6.143271
–	379	COC1=C(C=C(Br)C2=C1C=CC=C2)C(=O)N[C@H]1CCN(C1)C1C2CCCC1CCC2	6.863
–	373	OC12C3C4C5C3C(C3C5CC4C13)N2CC1CCCCC1	8.657577
–	369	C(CN1CCN(CC1)C1CCCCC1)CC1=CC=CC2=C1C=CC=C2	9.161151
–	366	O[C@H]1CCCC[C@@H]1N1C2CCC1CC(C2)C1=CC=CC=C1	6.928118
–	363	COC1=CC=CC2=C1CCCC2CCCCCN1CCC(CC1)C1CCCCC1	7.140862
–	362	CN1C(=O)N(CCCCN2CCN(CC2)C2=CC=C(F)C=C2)C2=CC(=CC=C12)[N+]([O-])=O	8.326058
–	361	CC(C1(CCN(CC1)[C@H]2[C@@H](CC[C@@H](C2)OCC3=CC=C(C=C3)F)O)C4=CC=CC=C4)=O	7.725842
–	358	FC1=CC(CCN2C3CCC2CC3)=CC=C1	7.530178
–	356	COC1=CC=C(CCN2C3CCCC2CC(C3)OC(=O)NC2=C(OC)C=CC(C)=C2)C=C1	6.900319
–	354	CCN(CCCCC1=CN(C2=C1C=CC=C2)C1=CC=C(F)C=C1)[C@@H]1CCC2=C1C=CC(OC)=C2OC	7.453457
–	347	CC(=O)C1=CC=C2OC(=O)N(CCCCN3CCN(CC3)C3CCCCC3)C2=C1	7.982549
–	345	COC1=C(OC)C(=CC(Br)=C1)C(=O)NCCCCN1CCC2=CC3=C(OCO3)C=C2C1	7.68403
–	344	CN1CCC2(CC1/C(=C/C1=CC=C(Cl)C=C1)C(=O)C2)C1=CC(O)=CC=C1	8.022276
–	343	CC(=O)C1=CC=C2N(CCCCN3CCN(CC3)C3=CC=C(F)C=C3)C(=O)COC2=C1	8.198
–	342	FC1=CC=C(C=C1)C1=CN(CCCCN2CCN(CC2)C2CCCCC2)C2=CC=CC=C12	7.646853
–	340	[H]N(CC1=CC=CC=C1)C1C2C3CC4C5CC(C2C35)C14	7.721246
–	338	COC1=CC2=C(CN(CC3CCN(CC3)C(=O)C3=CC=CC4=C3C=CC=C4)CC2)C=C1OC	6.939
–	331	COC1=CC=CC2=C1CCCC2CCCCCN1CCN(CC1)C1CCCCC1	9.455932
–	329	CC(=O)C1=CC=C2N(CCCCN3CCC(=CC3)C3=CC=C(F)C=C3)C(=O)OC2=C1	9.180456
–	328	COC1=C(NC(=O)OC2CC3CCCC(C2)N3CC2=CC=C(F)N=C2)C=C(C)C=C1	6.242984
–	324	[O-][N+](=O)C1=CC=C2N(CCCCN3CCN(CC3)C3CCCCC3)C(=O)OC2=C1	8.609
–	320	O=C(C1CCN(C2CC3=C(C(O)=CC=C3)CC2O)CC1)C4=CC=C(F)C=C4	5.732594
–	318	C[C@H](OC1=CC=C(F)C=C1)C(=O)OC1CC2CCC(C1)N2C	7.119186
–	315	C(NC1C2C3CC4C5CC(C2C35)C14)C1=CC=CN=C1	6.979
–	313	COC1=CC(OC)=CC(CN2C3C4C5C6C4C2(O)C2C6CC5C32)=C1	6.90309
–	310	COC1=CC=CC(=C1)[C@@H]1CC[C@@H](CC1)N1CCN(CC1)C1=NC=CC=C1	7.442493
–	307	CCC(SC1=CC=CC=C1)C(=O)OC1CC2CCC(C1)N2C	6.704213
–	306	O[C@@]12[C@H]3[C@@H]4[C@@H]5[C@H]3[C@@H]([C@H]3[C@@H]5C[C@@H]4[C@@H]13)N2CC1=CC=CC(Br)=C1	7.39794
–	302	C(CC1=CC=CC=C1)NC1C2C3CC4C5CC(C2C35)C14	8.031517
–	300	[H]C1(CC2CCC(C1)N2CC1=CC=CC=C1)OC(=O)NC1=CC(=CC=C1C)[N+]([O-])=O	8.509
–	298	CC1(C)C2CC[C@]1(C)CN(CCC1C3CC4CC(C3)CC1C4)C2	9.113509
–	291	COC(=O)[C@@]1(C[C@@H]1CNC12CC3CC(CC(C3)C1)C2)C1=CC=CC=C1	7.405
–	286	O[C@@]12[C@H]3[C@@H]4[C@@H]5[C@H]3[C@@H]([C@H]3[C@@H]5C[C@@H]4[C@@H]13)N2CC1=CC=CC(Cl)=C1	7.523
–	285	OC(CN1C2CCC1CC(C2)C1=CC=CC=C1)CC1=CC=C(Br)C=C1	8.305395
–	282	COC1=CC2=C(CN(CCCCNC(=O)C3=CC(Br)=CC(OC)=C3OC)CCC2)C=C1OC	6.501689
–	280	COC1=C(NC(=O)OC2CC3CCCC(C2)N3CC2=CC=C(F)C=C2)C=C(C)C=C1	7.522879
–	279	COC1=CC2=C(CCN(CCCCNC(=O)C3=CC(Br)=CC(OC)=C3OC)CC2)C=C1OC	6.134304
–	278	COC1=C2CCCC(C(=O)NCCCN3CCC4=CC(OC)=C(OC)C=C4C3)C2=CC=C1	7.676
–	270	COC1=CC=C(CN2C3C4C5C6C4C2(O)C2C6CC5C32)C=C1	7.563837
–	268	[O-][N+](=O)C1=CC=C(C=C1)C(=O)C1=CC=C(C=C1)C(=O)NCCCN1CCN(CCCNCC#C)CC1	7.220837
–	266	COC1=CC2=C(CCCC2CCCN2CCN(CC2)C2CCCCC2)C=C1	8.91364
–	259	COC1=CC2=C(CCN(CCCCCNC(=O)C3=CC4=C(O3)C=CC(I)=C4)C2)C=C1	7.508638
–	252	[O-][N+](=O)C1=CC=C(NCCCCCCCCCCOC2=CC=CC3=C2CCCC3CCCN2CCN(CC2)C2CCCCC2)C2=NON=C12	7.505845
–	250	CN1CC2(CCN(C[C@H](O)CC3=CC=C(Br)C=C3)CC2)C2=CC=CC=C12	7.640165
–	249	O[C@@H](CN1CCC2(CCC3=CC=CC=C23)CC1)C1=CC(Br)=CC=C1	7.896196
–	248	C(CCN1CCC2(CC1)OCC1=C2C=CC=C1)CN1C2=CC=CC=C2C2=C1C=CC=C2	8.489455
–	247	O=C(C1=CC=CC=C1)C1=CC=C(C=C1)N(CCCN1CCN(CC1)C1CCCCC1)CC#C	7.113509
–	242	CC(C(=O)OC1CC2CCC(C1)N2C)C1=CC=C(Cl)C=C1	6.482804
–	241	COC1=C(OC)C=C2CN(CCNC(=O)C3=C(OC)C4=CC=CC=C4C(Br)=C3)CCC2=C1	7.673664
–	240	CN1C2CCC1CC(C2)OC(C1=CC=CC=C1)C1=CC=CC=C1	7.458
–	236	COC1=CC2=C(CN(CCCCN3C=C(CCCF)C4=C3C=CC=C4)CC2)C=C1OC	7.545155
–	235	COC1=C(OC)C=C(CN(C)C2C3C4CC5C6CC(C3C46)C25)C=C1	7.568636
–	233	COC1=C(NC(=O)OC2CC3CCCC(C2)N3CCC2=CC=C(C=C2)N(C)C)C=C(C)C=C1	6.70952
–	229	FC1=CC(CCCN2C3CCC2CC3)=CC=C1	7.924
–	228	OC1CN(CCC2=CC(O)=CC=C12)C1CCN(CC1)C(=O)C1=CC=CC=C1	7.008774
–	225	FC1=CC=CC(CNC2C3C4CC5C6CC(C3C46)C25)=C1	7.721246
–	224	COC1=CC2=C(NC=C2C(=O)CN2CCC(O)(CC2)C2=CC=C(Cl)C=C2)C=C1	8.124939
–	223	O=C1SC2=C(C=CC=C2)N1CCCCN1CCN(CC1)C1CCCCC1	9.409
–	222	COC1=CC2=C(CN(CCCCNC(=O)C3=CNN=N3)CC2)C=C1OC	5.990124
–	217	O=C(N)C1=CC=C(N2N=C(C)C3=C2CC(C)(C)CC3=O)C=C1NCCCN4CC5=C(C=C(OC)C(OC)=C5)CC4	7.046724
–	216	COC1=CC2=C(C=C1)C(CCCN1CCCCC1(C)C)=CC=C2	7.026872
–	213	CCC(SC1=CC=C(Cl)C=C1)C(=O)OC1CC2CCC(C1)N2C	7.070581
–	212	OC1=CC2=C(NC=C2C(=O)CN2CCC(O)(CC2)C2=CC=C(Cl)C=C2)C=C1	7.328
–	210	OC1=CC2=C(NC=C2C(=O)CN2CCC(CC2)C2=CC=CC=C2)C=C1	6.649752
–	209	COC1=C(NC(=O)OC2CC3CCCC(C2)N3CCC2=CC=C(C)C=C2)C=C(C)C=C1	7.080922
–	205	OC(CN1C2CCC1CC(C2)C1=CC=CC=C1)C1=C(Br)C=CC=C1	8.112946
–	199	O=C(N)C1=CC=C(N2N=C(C)C3=C2CC(C)(C)CC3=O)C=C1NCCCCCN4CC5=C(C=C(OC)C(OC)=C5)CC4	7.127844
–	196	CC(SC1=CC=C(Br)C=C1)C(=O)OC1CC2CCC(C1)N2C	6.493495
–	196	CN(CCC1=CC=CC=N1)C1C2C3CC4C5CC(C2C35)C14	7.49485
–	194	CC1=CC=CC=C1[C@@H]1CC[C@@H](CC1)N1CCN(CC1)C1=NC=CC=C1	7.148742
–	191	FC1=CC=C2N(CCCCN3CCN(CC3)C3CCCCC3)C(=S)SC2=C1	9.301
–	190	FC1=CC=C(C=C1)N1CCN(CCCCN2C=C(C3=CC=CC=C23)C2=CC=C(F)C=C2)CC1	7.079199
–	189	COC1=C(NC(=O)OC2CC3CCCC(C2)N3CC=C)C=C(C)C=C1	7.066
–	188	COC1=C(NC(=O)OC2CC3CCCC(C2)N3CC2=CC=C(I)C=C2)C=C(C)C=C1	7.514279
–	184	COC1=C(OC)C(=CC(Br)=C1)C(=O)NCCCCN1CCC2=CC3=C(OCCO3)C=C2C1	7.664
–	179	COC1=CC=C(CNCCCCC2=CN(C3=C2C=CC=C3)C2=CC=C(F)C=C2)C=C1OC	6.772113
–	174	CC(CCCN1C(=O)CSC2=CC=CC=C12)N1CCN(CC1)C1CCCCC1	8.263603
–	173	COC1=CC2=C(CN(CCCCCNC(=O)C3=CC4=C(O3)C=CC(I)=C4)CC2)C=C1	8.356547
–	172	COC1=CC2=C(CN(CCC3CCN(CC3)C(=O)C3=CC4=C(O3)C=CC(I)=C4)CC2)C=C1OC	7.975
–	169	COC1=CC2=C(CN(CCCCN3C=C(CCF)C4=C3C=CC=C4)CC2)C=C1OC	7.560667
–	167	C(CCN1CCC(CC1)C1CCCCC1)CN1C2=CC=CC=C2C2=C1C=CC=C2	8.116
–	166	CCC(OC1=CC=C(Cl)C=C1)C(=O)OC1CC2CCC(C1)N2C	7.171
–	165	COC1=C(NC(=O)OC2CC3CCCC(C2)N3CCC2=CC=C(C=C2)[N+]([O-])=O)C=C(C)C=C1	7.939
–	159	C(CN1C=CC2=C1C=CC=C2)CN1CCN(CC1)C1=CC=CC=C1	7.124939
–	156	COC1=C(NC(=O)OC2CC3CCCC(C2)N3CC2=CC=C(C=C2)[N+]([O-])=O)C=C(C)C=C1	7.598599
–	153	CN(CCCCC1=CN(C2=C1C=CC=C2)C1=CC=C(F)C=C1)CC1=CC=C(C)C=C1C	8.229
–	151	OC1CN(CCC2=CC(O)=CC=C12)[C@H]1CC[C@@H](CC1)C1=CC=CC=C1	8.05061
–	149	FC1=CC=C(C=C1)N1CCN(CCCN2C=CC3=C2C=CC=C3)CC1	7.699
–	148	COC1=CC2=C(CN(CCCCCNC(=O)C3=CC4=C(O3)C=CC(I)=C4)CC2)C=C1OC	8.05061
–	147	CC(=O)C1=CC=C2OC(=O)N(CCCCN3CCN(CC3)C3=CC=C(F)C=C3)C2=C1	8.917
–	146	NC1=C2C=CC=C3C(=O)N(CCCCCCOC4=CC=CC5=C4CCCC5CCCN4CCN(CC4)C4CCCCC4)C(=O)C(C=C1)=C23	8.182435
–	140	O[C@@]12[C@H]3[C@@H]4[C@@H]5[C@H]3[C@@H]([C@H]3[C@@H]5C[C@@H]4[C@@H]13)N2CC1=CC=CC=C1	7.920819
–	139	COC1=C(NC(=O)OC2CC3CCCC(C2)N3CCCCCCC2=CC=CC=C2)C=C(C)C=C1	8.119186
–	138	COC1=C2C=CC=CC2=C(Br)C=C1C(=O)NCCCCN1CCN(CC1)C1=CC=CC(Cl)=C1Cl	7.578396
–	137	OC1CN(CCCOC2=CC=CC=C2)CCC2=CC(O)=CC=C12	8.229148
–	136	CN(CCC1=CC=CC(F)=C1)C1C2C3CC4C5CC(C2C35)C14	7.408935
–	133	COC1=C(NC(=O)OC2CC3CCCC(C2)N3CCCCCCCC2=CC=CC=C2)C=C(C)C=C1	8.119
–	127	COC1=CC2=C(CN(CCCC3CCN(CC3)C(=O)C3=CC4=C(O3)C=CC(Br)=C4)CC2)C=C1OC	8.276
–	126	COC1=C(OC)C=C2CN(CCCCNC(=O)C3=CC(Br)=CC(OC)=C3OCCF)CCC2=C1	6.413413
–	123	COC1=CC=CC2=C1CCCC2CCCN1CCN(CCCC2CCCC3=C(OC)C=CC=C23)CC1	6.920819
–	118	COC1=C(NC(=O)OC2CC3CCCC(C2)N3CCC2=CC=C(F)C=C2)C=C(C)C=C1	8.229
–	114	COC1=C(O)C(=CC=C1)C(=O)NCCCCN1CCC2=CC(OC)=C(OC)C=C2C1	5.853872
–	113	COC1=C(OC)C=C2CN(CCCCN3C(=O)OC4=CC=CC=C34)CCC2=C1	8.818
–	112	COC1=C(NC(=O)OC2CC3CCCC(C2)N3CCC2=CC=CC=C2)C=C(C)C=C1	8.920819
–	111	COC1=C(OC)C=C(C(=O)NCCCN2CCC3=CC(OC)=C(OC)C=C3C2)C(Br)=C1	7.293282
–	108	COC1=C(NC(=O)OC2CC3CCCC(C2)N3CCCCNCC2=CC=CC(I)=C2)C=C(C)C=C1	8.917215
–	107	NC1=CC=C2N(CCCCN3CCN(CC3)C3=CC=C(F)C=C3)C(=O)OC2=C1	6.750875
–	105	COC1=C(NC(=O)OC2CC3CCCC(C2)N3C/C=C/C2=CC=C(N)C=C2)C=C(C)C=C1	8.443697
–	97	COC1=CC=C2C(=O)N(CCCN3CCC4=CC(OC)=C(OC)C=C4C3)CCC2=C1	8.241088
–	93	COC1=CC2=C(CN(CCC3CCN(CC3)C(=O)C3=CC4=C(O3)C=CC(Br)=C4)CC2)C=C1OC	8.31
–	92	COC1=C(OC)C=C2CN(CCCCNC(=O)C3=CC=C(C=C3)C3=CN(CCOCCOCCF)N=N3)CCC2=C1	6.673664
–	91	COC1=C(NC(=O)OC2CC3CCCC(C2)N3CCCCCCCCCCNS(=O)(=O)C2=CC=CC3=C2C=CC=C3N(C)C)C=C(C)C=C1	7.853872
–	89	COC1=C(NC(=O)OC2CC3CCCC(C2)N3CCCCCCNS(=O)(=O)C2=CC=CC3=C2C=CC=C3N(C)C)C=C(C)C=C1	6.829738
–	84	COC1=CC2=C(CN(CC3CCN(CC3)C(=O)C3=CC4=CC(Br)=CC=C4N3)CC2)C=C1OC	7.398
–	76	COC1=CC=CC=C1CN1C=C(N=N1)C(=O)NCCN1CCC2=CC(OC)=C(OC)C=C2C1	6.806875
–	71	[H][C@@]12CS[C@H](CCCCC(=O)NCCCCCCCCCCN3C4CCCC3CC(C4)OC(=O)NC3=C(OC)C=CC(C)=C3)[C@]1([H])NC(=O)N2	7.156767
–	65	COC1=C(OC)C=C2CN(CCNC(=O)C3=CC(Br)=CC(OC)=C3OC)CCC2=C1	7.785
–	62	COC1=C(OC)C=C2CN(CCCCN3C(=O)C4=C(C=C(F)C=C4)C3=O)CCC2=C1	8.09691
–	60	COC1=C(OC)C=C2CN(CCNC(=O)C3=CC(C)=CC=C3OCCF)CCC2=C1	6.9914
–	58	CN1CC[C@]2(C[C@@H]1/C(=C/C1=CC=C(Cl)C(Cl)=C1)C(=O)C2)C1=CC(O)=CC=C1	7.873
–	54	COC1=C(NC(=O)OC2CC3CCCC(C2)N3CCCCCCN)C=C(C)C=C1	8.284833
–	53	C(CN1C2=C(C=CC=C2)C2=C1C=CC=C2)CN1CCN(CC1)C1CCCCC1	7.899629
–	51	COC1=C(OC)C=C2CN(CCCCNC(=O)C3=C(OCC#C)C=CC(=C3)C#N)CCC2=C1	7.91364
–	45	COC1=CC2=C(CN(CCCCNC(=O)C3=CN(CC4=CC=CC=C4)N=N3)CC2)C=C1OC	7.906578
–	44	COC1=CC(CN2C=C(N=N2)C(=O)NCCN2CCC3=CC(OC)=C(OC)C=C3C2)=CC=C1	6.669586
–	38	COC1=C(OC)C=C2CN(CCCCNC(=O)C3=C(OCC4=CN(CCF)N=N4)C=CC(=C3)C#N)CCC2=C1	7.496209
–	35	COC1=C(NC(=O)OC2CC3CCCC(C2)N3CCC2=CC=C(N)C=C2)C=C(C)C=C1	8.30103
–	34	COC1=C(OC)C=C2CN(CCCN3CCC4=C(C=CC=C4OCCCCCCN4C(=O)C5=C(C=C(C=C5)N(C)C)C4=O)C3=O)CCC2=C1	7.9914
–	31	COC1=C(NC(=O)OC2CC3CCCC(C2)N3CC2=CC=C(C=C2)N(C)C)C=C(C)C=C1	8.58838
–	30	COC1=C2CCN(CCCN3CCC4=CC(OC)=C(OC)C=C4C3)C(=O)C2=CC=C1	8.372634
–	28	COC1=CC=C(CN2C=C(N=N2)C(=O)NCCCCN2CCC3=C(C2)C=C(OC)C(OC)=C3)C=C1	8.30103
–	26	COC1=CC2=C(CN(CCCCNC(=O)C3=CN(N=N3)C3=CC=CC=C3OCCF)CC2)C=C1OC	7.619789
–	24	COC1=CC2=C(CN(CCCCNC(=O)C3=CN(CC4=CC=CC(OCCF)=C4)N=N3)CC2)C=C1OC	7.206908
–	21	CN1C(=O)N(CCCCN2CCN(CC2)C2=CC=C(F)C=C2)C2=CC=CC=C12	9.337242
–	19	C(CNC1(CC2CCC1C2)C1=CC=CC=C1)CN1CCCCC1	8.018
–	18	COC1=C(OC)C=C2CN(CCCCN3C=C(C4=C3C=CC=C4)C3=CC=C(F)C=C3)CCC2=C1	8.359519
–	12	FC1=CC=C(C=C1)C1(CC2CCC1C2)NCCCOC1CCCCO1	8.259637
–	11	COC1=C(OC)C=C2CN(CCCCNC(=O)C3=CC=CC=C3)CCC2=C1	5.276544
–	10	COC1=C(NC(=O)OC2CC3CCCC(C2)N3CC2=CC=C(CCF)C=C2)C=C(C)C=C1	9.086186
–	8	COC1=CC=CC=C1CN1C=C(N=N1)C(=O)NCCCCN1CCC2=C(C1)C=C(OC)C(OC)=C2	8.013228
–	7	FC1=CC=C(C=C1)C1=CC=C2OC(=O)N(CCCCN3CCN(CC3)C3CCCCC3)C2=C1	11.21467
–	5	COC1=CC=CC(=C1)N1C=C(N=N1)C(=O)NCCCCN1CCC2=C(C1)C=C(OC)C(OC)=C2	8.522879
+	2	COC1=CC2=C(CN(CCCCNC(=O)C3=CN(N=N3)C3=CC=CC=C3OC)CC2)C=C1OC	8.823909
+	3	COC1=C(OC)C=C2CN(CCCCNC(=O)C3=CC(I)=CC(OC)=C3OCCF)CCC2=C1	9.585027
+	4	COC1=CC=C(C=C1)N1C=C(N=N1)C(=O)NCCCCN1CCC2=CC(OC)=C(OC)C=C2C1	8.259637
+	6	COC1=CC2=C(CN(CCCCNC(=O)C3=CN(CC4=CC=C(O)C=C4)N=N3)CC2)C=C1OC	7.935542
+	9	COC1=CC2=C(CN(CCCCNC(=O)C3=CN(N=N3)C3=CC=C(OCCF)C=C3)CC2)C=C1OC	7.66
+	13	COC1=C(OC)C=C2CN(CCCCNC(=O)C3=CC(Br)=CC(OC)=C3OC)CC2=C1	9.086186
+	14	COC1=C(OC)C=C2CN(CCCCNC(=O)C3=CC(Br)=CC=C3OCCF)CCC2=C1	9.187087
+	15	COC1=C(OC)C=C2CN(CCCCNC(=O)C3=CC(Br)=CC(OC)=C3OC)CCC2=C1	8.086186
+	16	COC1=C(OC)C=C2CN(CCCCNC(=O)C3=CC(I)=CC=C3OCCF)CCC2=C1	8.974694
+	22	COC1=CC2=C(CN(CCCCCCNC(=O)C3=CC4=C(O3)C=CC(I)=C4)CC2)C=C1OC	9.055517
+	23	COC1=CC=C(C)C=C1C(=O)NCCN1CCC2=CC(OC)=C(OC)C=C2C1	7.876148
+	27	O=C1N(CCCCN2CCN(C3=CC=C(F)C=C3)CC2)C4=C(C=C(N=C=S)C=C4)O1	7.835647
+	32	COC1=CC2=C(CN(CCCCNC(=O)C3=CC=C(C=C3)C3=CN(CCF)N=N3)CC2)C=C1OC	6.857
+	36	COC1=CC2=C(CN(CCCCNC(=O)C3=CN(CC4=CC=C(OCCF)C=C4)N=N3)CC2)C=C1OC	7.316953
+	39	COC1=CC=CC(=C1)N1C=C(N=N1)C(=O)NCCN1CCC2=CC(OC)=C(OC)C=C2C1	6.744727
+	41	COC1=C(OC)C=C2CN(CCCCNC(=O)C3=C(OC)C=CC(=C3)N3C=C(CN(C)C)N=N3)CCC2=C1	7.246
+	42	COC1=C(NC(=O)OC2CC3CCCC(C2)N3CCCCCCNC(=O)C2=CC=C(Cl)C=C2)C=C(C)C=C1	8.44855
+	43	COC1=CC=C(C(=O)NCCCCN2CCC3=CC=C(C=C3C2)[N+]([O-])=O)C(OC)=C1OC	7.571865
+	47	COC1=C(OC)C=C2CN(CCCCN3C=CC4=C3C=CC=C4)CCC2=C1	9.677781
+	48	COC1=CC(Br)=CC(C(=O)NCCCN2CCC3=CC(OC)=C(OC)C=C3C2)=C1OC	7.88941
+	49	COC1=C(OC)C=C2CN(CCCN3CCC4=C(OCCF)C=CC=C4C3=O)CCC2=C1	8.034
+	50	COC1=C(C=C(C=C1)N1C=C(N=N1)C1=CN=CN1C)C(=O)NCCCCN1CCC2=CC(OC)=C(OC)C=C2C1	7.68403
+	52	COC1=CC2=C(CN(CC3CCN(CC3)C(=O)C3=CC4=C(O3)C=CC(Br)=C4)CC2)C=C1OC	8.06
+	55	COC1=CC2=C(CN(CCCCNC(=O)C3=CC4=C(O3)C=CC(Br)=C4)CC2)C=C1OC	7.823909
+	56	COC1=CC=C(C)C=C1C(=O)NCCCCN1CCC2=CC(OC)=C(OC)C=C2C1	8.06148
+	57	COC1=CC=C(C=C1)N1C=C(N=N1)C(=O)NCCN1CCC2=CC(OC)=C(OC)C=C2C1	6.387216
+	59	COC1=CC2=C(CN(CCCCNC(=O)C3=CC(=CC=C3OCCF)N3C=C(CN(C)C)N=N3)CC2)C=C1OC	7.026872
+	61	COC1=C(C=C(C)C=C1)C(=O)NCCCN1CCC2=CC(OC)=C(OC)C=C2C1	8.580044
+	68	COC1=CC(/C=C2/[C@H]3C[C@](CCN3C)(CC2=O)C2=CC(O)=CC=C2)=CC=C1	7.417937
+	69	COC1=CC=CC=C1CN1C=C(N=N1)C1=CC=C(C=C1)C(=O)NCCCCN1CCC2=CC(OC)=C(OC)C=C2C1	7.259637
+	70	FC1=CC=C(C=C1)N1CCN(CCCCN2C=CC3=CC=CC=C23)CC1	8.962574
+	72	COC1=C(OC)C=C2CN(CCCN3CCC4=CC=CC=C4C3=O)CCC2=C1	8.315155
+	78	COC1=C(OC)C=C2CN(CCCCCN3C(=O)C4=C(C=C(F)C=C4)C3=O)CCC2=C1	8.619789
+	81	COC1=CC2=C(CN(CCNC(=O)C3=CN(N=N3)C3=CC=CC=C3OC)CC2)C=C1OC	6.823909
+	83	OC1=CC2=C(C=C1)C(=CN2)C(=O)CN1CCC(CC2=CC=CC=C2)CC1	7.367
+	85	COC1=CC2=C(NC=C2C(=O)CN2CCC(CC2)C2=CC=CC=C2)C=C1	7.69897
+	87	COC1=C(OC)C=C2CN(CCCN3CCC4=CC(F)=CC=C4C3=O)CCC2=C1	8.44855
+	88	COC1=C(O)C(=CC=C1I)C(=O)NCCCCN1CCC2=CC=C(C=C2C1)[N+]([O-])=O	6.062
+	90	CN1CC[C@]2(C[C@@H]1/C(=C/C1=CC=CC=C1)C(=O)C2)C1=CC(O)=CC=C1	7.782516
+	95	[O-][N+](=O)C1=CC=C(C=C1)C(=O)C1=CC=C(CNCCCN2CCN(CC2)C2=CC=C(F)C=C2)C=C1	7.468904
+	96	CC(O)C1=CC=C2N(CCCCN3CCN(CC3)C3=CC=C(F)C=C3)C(=O)OC2=C1	8.06148
+	98	CN1CC[C@]2(C[C@@H]1/C(=C/C1=CC=CC(I)=C1)C(=O)C2)C1=CC(O)=CC=C1	7.62
+	99	COC1=CC2=C(CN(CCCCCNC(=O)C3=CC4=C(O3)C=CC(Br)=C4)CC2)C=C1OC	8.207608
+	101	FC1=CC=C(C=C1)N1CCN(CCCCN2C(=O)NC3=CC=CC=C23)CC1	7.529
+	102	COC1=CC2=C(CN(CC3CCN(CC3)C(=O)C3=CC4=C(O3)C=CC(I)=C4)CC2)C=C1OC	7.958607
+	103	OC1=CC2=C(NC=C2C(=O)CN2CCC(CC3=CC=CC=C3)CC2)C=C1	7.116
+	109	OC12C3C4C5C3C(C3C5CC4C13)N2CC1=CC=CC=N1	7.69897
+	110	COC1=C(OC)C=C2CN(CCCCN3C=C(C4=COC=C4)C4=C3C=CC=C4)CCC2=C1	7.920096
+	116	COC1=C(OC)C=C2CN(CCCCNC(=O)C3=CC(C)=CC=C3OCCF)CCC2=C1	8.158
+	120	COC1=C(OC)C(=CC(Br)=C1)C(=O)NCCCCN1CCC2=CC3=C(OCCCO3)C=C2C1	7.486782
+	121	OC12C3C4C5C3C(C3C5CC4C13)N2CCC1=CC=CC=N1	7.571865
+	122	[H][C@@]12C[C@H]3C[C@H](CC)[C@]1([H])N(C3)CCC1=C2NC2=C1C=C(OC)C=C2	6.696804
+	124	COC1=C(NC(=O)OC2CC3CCCC(C2)N3CCCCCCCCCCNC2=CC=C(C3=NON=C23)[N+]([O-])=O)C=C(C)C=C1	7.958607
+	125	[O-][N+](=O)C1=CC=C(C=C1)C(=O)C1=CC=C(CNCCCN2CCN(CC2)C2CCCCC2)C=C1	7.477
+	128	CN(CCCC1=CC=CC(F)=C1)C1C2C3CC4C5CC(C2C35)C14	7.958607
+	129	COC1=C(OC)C=C2CN(CC3=CC=C(N3)C3=C(OC)C4=CC=CC=C4C(Br)=C3)CCC2=C1	7.585027
+	131	CN1CC[C@]2(C[C@@H]1/C(=C/C1=CC=C(I)C=C1)C(=O)C2)C1=CC(O)=CC=C1	7.279
+	132	OC1CN(CCCC(=O)C2=CC=C(F)C=C2)CCC2=CC(O)=CC=C12	7.481
+	134	[H][C@@]12CS[C@H](CCCCC(=O)NCCCCCCN3C4CCCC3CC(C4)OC(=O)NC3=C(OC)C=CC(C)=C3)[C@]1([H])NC(=O)N2	6.943
+	135	COC1=C(NC(=O)OC2CC3CCCC(C2)N3CC2=CC=CC=C2)C=C(C)C=C1	8.508638
+	141	FC1=C(C=CC=C1)N1CCN(CCCN2C=CC3=C2C=CC=C3)CC1	8.004365
+	142	COC1=C(NC(=O)OC2CC3CCCC(C2)N3CC2=CC=CC(I)=C2)C=C(C)C=C1	7.293282
+	144	COC1=C(NC(=O)OC2CC3CCCC(C2)N3CCCCCCNC2=CC=C(C3=NON=C23)[N+]([O-])=O)C=C(C)C=C1	7.347
+	150	COC1=C(NC(=O)OC2CC3CCCC(C2)N3CCCCC2=CC=CC=C2)C=C(C)C=C1	8.538
+	152	FC1=CC=C(C=C1)N1CCN(CCCCN2C=C(C3=CC=CC=C23)C2=CC=CC=C2)CC1	8.001741
+	154	COC1=C(NC(=O)OC2CC3CCCC(C2)N3CCCCNCC2=CC=CC(Br)=C2)C=C(C)C=C1	8.514279
+	157	IC1=CC=C(C=C1)N1C=C(CCCCN2CCC(CC2)(C#N)C2=CC=CC=C2)C2=C1C=CC=C2	7.732828
+	158	COC(=O)[C@]1(C[C@H]1CN1C2CCC1CC(O)(C2)C1=CC=C(Cl)C=C1)C1=CC=CC=C1	7.599
+	160	COC1=CC2=C(CN(CCCCCCNC(=O)C3=CC4=C(O3)C=CC(Br)=C4)CC2)C=C1OC	8.097
+	170	COC1=C(NC(=O)OC2CC3CCCC(C2)N3CC2=CC=C(C)C=C2)C=C(C)C=C1	7.809668
+	175	C(CN1CCN(CC1)C1=CC=CC=N1)CC1=CC=CC=C1	8.308919
+	176	COC1=CC=CC2=C1CCCC2CCCN1CCN(CC1)C(=O)C1CCCCC1	6.517126
+	177	COC1=C(OC)C=C2CN(CCCCN3C(=S)SC4=CC=CC=C34)CCC2=C1	9.252
+	183	COC1=C(NC(=O)OC2CC3CCCC(C2)N3CC2=CC=CC=C2)C=CC(Cl)=C1	7.197226
+	185	COC1=C(NC(=O)OC2CC3CCCC(C2)N3CCCC2=CC=C(N)C=C2)C=C(C)C=C1	7.979
+	186	COC1=CC2=C(CN(CCCCN3C=C(C4=CC=CC=C34)C3=CC=CC=C3)CC2)C=C1OC	7.334513
+	192	COC1=C(C=C(Br)C2=C1C=CC=C2)C(=O)NC1CC2CCCC(C1)N2C1CCCCC1	8.318759
+	193	OC1CN(CCC2=CC(O)=CC=C12)[C@H]1CC[C@H](CC1)C1=CC=CC=C1	8.065502
+	197	FC1=CC=C(C=C1)N1C=C(CCCCN2CCC(CC2)C2CCCCC2)C2=CC=CC=C12	8.138
+	200	OC(CN1C2CCC1CC(C2)C1=CC=CC=C1)C1=CC(Cl)=C(Cl)C=C1	7.725842
+	201	C1C[C@H](CC[C@H]1N1CCN(CC1)C1=NC=CC=C1)C1=CC=CC=C1	7.207608
+	203	CC(CCCN1C(=O)OC2=CC(=CC=C12)C(C)=O)N1CCN(CC1)C1=CC=C(F)C=C1	8.667562
+	204	COC1=C(NC(=O)OC2CC3CCCC(C2)N3CCCCCCNCC2=CC=CC(Br)=C2)C=C(C)C=C1	8.048662
+	206	COC1=CC(NC(=O)OC2CC3CCCC(C2)N3CC2=CC=CC=C2)=C(OC)C=C1	7.571865
+	207	COC1=CC(CN2C3C4C5C6C4C2(O)C2C6CC5C32)=CC=C1	7.572
+	208	COC1=C(C=C(Br)C2=C1C=CC=C2)C(=O)NC1CC2CCCC(C1)N2C1CCCCCC1	8.102373
+	211	OC(CN1C2CCC1CC(C2)C1=CC=CC=C1)C1=CC(Br)=CC=C1	8.006
+	214	FC1=CC=CC(F)=C1[C@@H]1CC[C@@H](CC1)N1CCN(CC1)C1=NC=CC=C1	6.356547
+	219	FC1=CC=C(C=C1)N1CCN(CCCCN2C(=O)OC3=CC=CC=C23)CC1	9.154902
+	220	COC1=C(OC)C=C2CN(CCCCC3=CN(C4=C3C=CC=C4)C3=CC=C(F)C=C3)CCC2=C1	7.308035
+	226	COC1=CC2=C(CN(CCCCN3C=CC4=CC(=CC=C34)C(C)=O)CC2)C=C1OC	8.847712
+	230	CN(CCCCN(C)CCC1=CC=C(Cl)C(Cl)=C1)CCCCN1CCCC1	6.939302
+	232	COC1=CC2=C(CN(CCCCNC(=O)C3=CC4=C(O3)C=CC(I)=C4)CC2)C=C1	6.876148
+	234	COC1=C(NC(=O)OC2CC3CCCC(C2)N3CCC2=CC=C(CCF)C=C2)C=C(C)C=C1	6.559406
+	237	FC1=CC=C2N(CCCCN3CCN(CC3)C3CCCCC3)C(=O)SC2=C1	9.657577
+	238	CC(CCCN1C(=S)SC2=CC=CC=C12)N1CCN(CC1)C1CCCCC1	9.004
+	243	COC1=CC=CC(OC)=C1[C@@H]1CC[C@@H](CC1)N1CCN(CC1)C1=NC=CC=C1	6.163043
+	245	COC1=CC=CC(OC)=C1[C@H]1CC[C@@H](CC1)N1CCN(CC1)C1=NC=CC=C1	7.60206
+	246	COC1=C2CCCC(CCCCN3CCN(CC3)C3=NCCCC3)C2=CC=C1	7.332547
+	253	OC12C3C4C5C3C(C3C5CC4C13)N2CCC1=CC=CN=C1	7
+	254	O=C(CN(CCCN1CCN(CC1)C1CCCCC1)CC#C)NC1=CC=C(C=C1)C(=O)C1=CC=CC=C1	8.675718
+	255	COC1=CC=C(CN2C3CCCC2CC(C3)OC(=O)NC2=C(OC)C=CC(C)=C2)C=C1	7.39
+	256	COC1=CC2=C(CN(CCCC3CCN(CC3)C(=O)C3=CC4=C(O3)C=CC(I)=C4)CC2)C=C1OC	7.906578
+	262	CN1C(=O)N(CCCCN2CCN(CC2)C2=CC=C(F)C=C2N=C=S)C2=CC=CC=C12	7.899629
+	264	FC1=CC=C(N2CCN(CCCCN3C(=O)OC4=CC=CC=C34)CC2)C(F)=C1	8.707744
+	265	C(CCN1CCN(CC1)C1CCCCC1)CN1C=C(C2=CC=CC=C12)C1=CC=CC=C1	7.910095
+	269	CN(CCC1=CC=CC(F)=C1)[C@@H]1C2C3C4C2C(=O)C2C4CC3C12	7.259637
+	272	[O-][N+](=O)C1=CC=C(NCCCCCCOC2=CC=CC3=C2CCCC3CCCN2CCN(CC2)C2CCCCC2)C2=NON=C12	7.966576
+	274	O=C(N)C1=CC=C(N2N=C(C)C3=C2CC(C)(C)CC3=O)C=C1NCCCCN4CC5=C(C=C(OC)C(OC)=C5)CC4	6.847712
+	276	[H]C1(CC2CCC(C1)N2CC1=CC=CC=C1)OC(=O)NC1=CC(=CC=C1OC)[N+]([O-])=O	8.267606
+	277	COC1=CC=CC=C1CN1C2C3C4C5C3C1(O)C1C5CC4C21	7.563837
+	281	CC(=O)C1=CC=C2N(CCCCN3CCN(CC3)C3CCCCC3)C(=O)OC2=C1	8.152427
+	288	COC1=CC2=C(NC=C2C(=O)CN2CCC(O)(CC2)C2=CC=C(Br)C=C2)C=C1	8
+	289	COC1=CC2=C(CN(CC3CCN(CC3)C(=O)/C=C/C3=CC=CC=C3)CC2)C=C1OC	6.863279
+	290	COC1=CC2=C(CN(CC3CCN(CC3)C(=O)C3=CC(Br)=CC(OC)=C3OC)CC2)C=C1OC	6.435334
+	292	CC1(C)C2CC[C@@]1(C)CN(CCC1CCCCC1)C2	9.638
+	295	C(CN1CCCCC1)CC1=CC=CC=C1	7.60206
+	297	FCCN1C=C(CCCCN2CCC3(CC2)OCC2=CC=CC=C32)C2=C1C=CC=C2	7.357
+	299	FC1=CC=C(C=C1)N1CCN(CC2=CNC3=C2C=CC=C3)CC1	6.180456
+	301	FCCOC1=CC(=CC=C1)N1CCN(CCCCC2=CN(C3=C2C=CC=C3)C2=CC=C(F)C=C2)CC1	7.315155
+	303	CN1CC2(CCN(C[C@H](O)C3=CC(Br)=CC=C3)CC2)C2=CC=CC=C12	7.003488
+	304	[H][C@@]12CS[C@H](CCCCC(=O)NCCCCCC(=O)NCCCCCCCCCCN3C4CCCC3CC(C4)OC(=O)NC3=C(OC)C=CC(C)=C3)[C@]1([H])NC(=O)N2~	6.135
+	305	CN(CCCN(C)CCC1=CC=C(Cl)C(Cl)=C1)CCCN1CCCC1	7.826814
+	309	O[C@@]12[C@H]3[C@@H]4[C@@H]5[C@H]3[C@@H]([C@H]3[C@@H]5C[C@@H]4[C@@H]13)N2CC1=CC=CC(F)=C1	7.509
+	311	COC1=CC2=C(NC=C2C(=O)CN2CCC(O)(CC2)C2=CC=CC=C2)C=C1	7.678
+	312	COC1=CC=CC2=C1CCCC2CCCNCC(=O)NC1CCCCC1	6.097997
+	314	COC1=C(NC(=O)OC2CC3CCCC(C2)N3CC2=CC=CC=C2F)C=C(C)C=C1	6.686133
+	316	CN(CCC1=CC=CC=C1)C1C2C3C4C2C(=O)C2C4CC3C12	6.764472
+	319	OC1=CC2=C(NC=C2C(=O)CN2CCC(O)(CC2)C2=CC=CC=C2)C=C1	6.181774
+	326	CN(CCCC1=CC(F)=CC=C1)[C@@H]1C2C3C4C2C(=O)C2C4CC3C12	7.523
+	332	[H]C1(CC2CCC(C1)N2CC1=CC=CC=C1)OC(=O)NC1=CC(Cl)=C(OC)C=C1OC	8.136677
+	335	C(CCN1CCN(CC1)C1CCCCC1)CCC1=CC=CC2=C1C=CC=C2	9.244125
+	336	CCC1=C(NC(=O)OC2CC3CCCC(C2)N3CC2=CC=CC=C2)C=C(C=C1)C(C)C	8.229148
+	337	[O-][N+](=O)C1=CC=C(C=C1)C(=O)C1=CC=C(CN(CCCN2CCN(CC2)C2=CC=C(F)C=C2)CC#C)C=C1	7.158453
+	339	[O-][N+](=O)C1=CC=C2CCN(CCCCN3C(=O)C4=C(C=CC=C4)C3=O)CC2=C1	7.672437
+	346	[H]C1(CC2CCC(C1)N2CC1=CC=CC=C1)OC(=O)NC1=CC=C(C=C1OC)[N+]([O-])=O	8.69897
+	350	COC1=C(NC(=O)OC2CC3CCCC(C2)N3CC2=CC=CC=C2)C=CC(=C1)[N+]([O-])=O	8.259637
+	351	ICCCCN(CCCN1CCN(CC1)C1CCCCC1)CC(=O)NC1=CC=C(C=C1)C(=O)C1=CC=CC=C1	7.349595
+	352	FC1=CC=C(C=C1)C(=O)CN1C2CCC1CC(C2)C1=CC=CC=C1	7.134718
+	355	COC1=C(OC)C2=C([C@H](N(CC)CCCCC3=CN(C4=CC=C(F)C=C4)C5=C3C=CC=C5)CC2)C=C1	7.453
+	359	C(CCN1CCN(CC1)C1CCCCC1)CN1C=C(C2=COC=C2)C2=CC=CC=C12	7.996971
+	365	CN1C(=O)N(CCCCN2CCN(CC2)C2=CC=C(F)C=C2)C2=CC(=CC=C12)N=C=S	7.521434
+	367	O[C@@H]1CC2=CC=CC=C2C[C@H]1N1CCC(CC1)C1=CC(I)=CC=C1	7.330683
+	371	OC12C3C4C5C3C(C3C5CC4C13)N2CC1=CC=CN=C1	5.49026
+	372	COC1=C(OC)C=C2CN(CC(=O)NCC3=CN(C4=C3C=CC=C4)C3=CC=C(F)C=C3)CCC2=C1	5.789681
+	375	COC1=C(NC(=O)OC2CC3CCCC(C2)N3CC2=CC=NC=C2)C=C(C)C=C1	6.282662
+	376	C(CCN1CCCC2=CC=CN=C12)CN1CCN(CC1)C1CCCCC1	8.785156
+	383	COC1=CC=CC2=C1CCCC2NCCN1CCN(CC1)C1CCCCC1	8.071
+	388	FC1=CC(CCCNC2C3C4CC5C6CC(C3C46)C25)=CC=C1	8.045757
+	389	CC(=O)NC1=CC=C2N(CCCCN3CCN(CC3)C3CCCCC3)C(=O)OC2=C1	7.111427
+	392	CC(C(=O)OC1CC2CCC(C1)N2C)C1=CC=C(Br)C=C1	6.211832
+	393	FC1=CC=C(C=C1)N1CCN(CN2C=C(CN3CCN(CC3)C3=CC=C(F)C=C3)C3=C2C=CC=C3)CC1	6
+	394	CC(OC1=CC=C(Br)C=C1)C(=O)OC1CC2CCC(C1)N2C	6.427942
+	400	O=C(CCCC[C@@H]1SCC2NC(=O)NC12)NCCCN(CCCN1CCN(CC1)C1CCCCC1)CC(=O)NC1=CC=C(C=C1)C(=O)C1=CC=CC=C1	7.062783
+	401	[H]C1(CC2CCC(C1)N2CC1=CC=CC=C1)OC(=O)NC1=CC=C(Cl)C=C1OC	7.996
+	404	COC1=C(NC(=O)OC2CC3CCCC(C2)N3CCCCCCNC(=O)C2=CC=C(F)C=C2)C=C(C)C=C1	6.303644
+	405	C(CC1=CC=CC=N1)NC1C2C3CC4C5CC(C2C35)C14	8.066
+	411	COC1=C(OC)C(=CC(Br)=C1)C1=NC=C(CN2CCC(CC3=CC=CC=C3)CC2)N1	6.385103
+	415	OCC1(CC1CNC12CC3CC(CC(C3)C1)C2)C1=CC=CC=C1	8.653647
+	417	COC1=CC(I)=CC(C(=O)NC2CC3CCCC(C2)N3CC2=CC=CC=C2)=C1OC	6.377475
+	424	FC1=CC=C(C=C1)C(=O)CCCN1C2CCC1CC(C2)C1=CC=CC=C1	7.814
+	426	COC1=C(OC)C=C(CN(C)[C@@H]2C3C4C5C3C(=O)C3C5CC4C23)C=C1	6.326979
+	427	COC(=O)C1(CC1CNC(C)C12CC3CC(CC(C3)C1)C2)C1=CC=CC=C1	7.344862
+	429	[H]C1(CC2CCC(C1)N2CC1=CC=CC=C1)OC(=O)NC1=CC=C(Br)C=C1	8.537602
+	430	FC1=CC=C(C=C1)N1CCN(CCCN2C(=O)C3=CC=CC=C3C2=O)CC1	6.129789
+	434	CC(=O)C1=CC=C2N(CC3CCCC(C3)N3CCN(CC3)C3=CC=C(F)C=C3)C(=O)OC2=C1	8.222573
+	435	C(CC1=CC=CC=C1)N1C2CCC1CC2	6.882729
+	439	[H]C1(CC2CCC(C1)N2CC1=CC=CC=C1)OC(=O)NC1=CC=C(CC)C=C1	7.917
+	440	CC(=O)C1=CC=C2N(CCCCN3CCCN(CC3)C3=CC=C(F)C=C3)C(=O)OC2=C1	7.295849
+	443	CC1(C)CCCN(CC2=CC3=CC=CC=C3O2)C1	5.742321
+	445	CCCCN1CCN(CC2=CC=C(C=C2)C2=CC=CC=C2)[C@@H](CCO)C1	6.638272
+	449	CC1=CC=C(C=C1)N1CCN(CCCCN2C(=O)OC3=CC=CC=C23)CC1	6.970535
+	456	O=C1SC2=C(C=CC=C2)N1CCCN1CCN(CC1)C1CCCCC1	8.516
+	457	CC(=O)NC1=CC=C2N(CCCCN3CCN(CC3)C3=CC=C(F)C=C3)C(=O)OC2=C1	7.317584
+	460	OCC[C@H]1CN(CC2CCCCC2)CCN1CC1=C2C=CC=CC2=CC=C1	7.796
+	465	FC1=CC=C(C=C1)N1CCN(CCCCN2C=C(C3=COC=C3)C3=C2C=CC=C3)CC1	6.8041
+	467	CC1(C)CCCN(CCC2CCCC3=CC=CC=C23)C1	7.386
+	468	BrC1=CC=C2N(CCCCN3CCN(CC3)C3CCCCC3)C(=O)OC2=C1	8.349692
+	470	COC1=C(C=C(C)C=C1)C(=O)NCCCN1CCN(CC1)C1CCCCC1	7.583359
+	471	C(CCN1CCN(CC1)C1CCCCC1)CN1C=CC2=CC=CC=C12	8.721
+	473	COC1=CC=CC(=C1)C12CCN(C)C(C1)/C(=C/C1=CC=CC=C1)C(=O)C2	7.087778
+	474	OC12C3C4C5C3C(C3C5CC4C13)N2CC1=CC=C2OCOC2=C1	6.5867
+	475	O=C(C1=CC=C(F)C=C1)CCCCN2CCC(CO)CC2	7.043
+	481	CSC1=CC=CC(NC(=O)OC2CC3CCCC(C2)N3CC2=CC=CC=C2)=C1	8.075721
+	483	OC(CN1C2CCC1CC(C2)C1=CC=CC=C1)C1=CC=C(Br)C=C1	7.947691
+	484	COC1=CC2=C(C=C1)C(CCCN1CCCCC1(C)C)CCC2	6.939302
+	488	OC1CN(CCCS(=O)(=O)C2=CC=CC=C2)CCC2=CC(O)=CC=C12	6.185087
+	489	OC1CN(CCC2=CC(O)=CC=C12)C1CCC2(CC1)OCCO2	6.181774
+	491	CC1(C)CCCN(CCCC2=C3C=COC3=CC=C2)C1	7.37366
+	504	FC1=CC(CNC23OC4C5C6C(C25)C2CC6C4C32)=CC=C1	5.784627
+	507	OC[C@@]1(C[C@@H]1CNC12CC3CC(CC(C3)C1)C2)C1=CC=CC=C1	8.055517
+	508	COC1=C(OC)C(=CC(Br)=C1)C1=NC=C(CN2CCC(CC2)C2=CC=CC=C2)N1	6.385103
+	510	COC1=CC=NC(=C1)N(CCCN1CCN(CC1)C1CCCCC1)C(C)=O	6.510042
+	512	COC1=CC=CC2=C1CCC[C@@H]2CCCN1CCN(CC1)C1CCCCC1	9.309804
+	513	O[C@H]1[C@@H](C[C@H](CC1)OCC2=CC=C(C=C2)F)N3CC4=C(CC3)C=CC=C4	7.338
+	514	COC1=C2CCCC(CCCN3CCN(CC3)C3=CC=CC(Cl)=C3)C2=CC=C1	7.226945
+	519	COC1=CC(CCN2CCN(CCCC3=CC=CC=C3)CC2)=CC=C1OCC4=CC=CC=C4	7.784
+	520	O[C@@H]1CCN(CC2=CC(I)=CC=C2)C[C@H]1N1CCC2(CCC3=CC=CC=C23)CC1	6.649752
+	521	COC1=C(NC(=O)OC2CC3CCCC(C2)N3CCC2=CC=C(I)C=C2)C=C(C)C=C1	6.850781
+	525	COC1=CC(Br)=CC(C(=O)NCCCN2CCN(CC2)C2CCCCC2)=C1OC	7.673664
+	530	CC1(C)CCCN(CCCCCN2C3=C(C=CC=C3)C3=C2C=CC=C3)C1	6.931814
+	531	CCC1=CC=C(NC(=O)OC2CC3CCCC(C2)N3CC2=CC=CC=C2)C=C1	7.835647
+	535	C(NC1C2C3CC4C5CC(C2C35)C14)C1=CC2=C(OCO2)C=C1	7.823909
+	537	CC(C)(C)C1=CC=C(C=C1)C1(CCCN2CCC3=CC(OCC4=CC=CC=C4)=CC=C3C(O)C2)OCC(C)(C)CO1	6.045757
+	541	O[C@@H]1CC2=C(C[C@H]1N3CCC(CC3)CC4=CC=CC=C4)C=CC=C2	7.222
+	543	COC(=O)C1(CC1CN(C)C12CC3CC(CC(C3)C1)C2)C1=CC=CC=C1	7.950782
+	544	CC1=CC=CC=C1[C@H]1CC[C@@H](CC1)N1CCN(CC1)C1=NC=CC=C1	7.528708
+	545	FC1=CC=C2N=C(SCCCCN3CCN(CC3)C3CCCCC3)SC2=C1	7.860121
+	550	O[C@H]1CC2=CC=CC=C2C[C@@H]1N1C2CCC1CC(C2)C1=CC=CC=C1	6.756962
#	100	COC1=C(OC)C=C2CN(CCCCNC(=O)C3=C(OC)C4=CC=CC=C4C(Br)=C3)CCC2=C1	7.754487
#	104	CN1C(=O)N(CCCCN2CCN(CC2)C2=C(N)C=C(F)C=C2)C2=CC=CC=C12	8.444906
#	115	CN(CCC1=CC=CC=C1)C1C2C3CC4C5CC(C2C35)C14	7.79588
#	119	OC(CN1C2CCC1CC(C2)C1=CC=CC=C1)C1=CC=CC(Br)=C1	7.910095
#	145	CC(F)C1=CC=C2N(CCCCN3CCN(CC3)C3=CC=C(F)C=C3)C(=O)OC2=C1	8.172631
#	155	O=C(N)C1=CC=C(N2N=C(C)C3=C2CC(C)(C)CC3=O)C=C1NC4CC(N5)CCCC5C4	7.331614
#	162	COC1=C(OC)C=C2CN(CC3=CC=C(N3)C3=CC(Br)=CC(OC)=C3OC)C(C)CC2=C1	7.283997
#	164	COC1=C(NC(=O)OC2CC3CCCC(C2)N3CCCCCCCCCCN)C=C(C)C=C1	8.150581
#	171	COC1=CC2=C(CCN(CCCCNC(=O)C3=CC4=C(O3)C=CC(I)=C4)C2)C=C1	7.455932
#	180	COC1=CC=CC2=C1CCC[C@@H]2NC(=O)CN1CCN(CC1)C1CCCCC1	8.227678
#	182	CN(CCC1=NC=CC=C1)C1C2C3C4C2C(=O)C2C4CC3C12	6.199
#	195	COC1=C(NC(=O)OC2CC3CCCC(C2)N3CCF)C=C(C)C=C1	6.844664
#	202	COC1=C(NC(=O)OC2CC3CCCC(C2)N3CC2=CC=CN=C2)C=C(C)C=C1	6.735
#	218	FC1=CC(CCNC2C3C4CC5C6CC(C3C46)C25)=CC=C1	8.221849
#	231	COC1=C(NC(=O)OC2CC3CCCC(C2)N3CCCCCC2=CC=CC=C2)C=C(C)C=C1	8.745
#	244	CC1(C)C2CC[C@]1(C)CN(CCC1CCCCCCC1)C2	9.481
#	25	COC1=CC2=C(CN(CCCCNC(=O)C3=CN(N=N3)C3=CC=CC(OCCF)=C3)CC2)C=C1OC	7.6
#	257	[O-][N+](=O)C1=CC=C(C=C1)C(=O)C1=CC=C(CN(CCCN2CCN(CC2)C2CCCCC2)CC#C)C=C1	8.422508
#	260	CC1(C)C2CC[C@]1(C)CN(CCC1CCCCC1)C2	9.553
#	263	COC1=CC=CC2=C1CCCC2CCCN1CCC(CC1)C1CCCCC1	7.726
#	271	COC1=CC2=C(CN(CC3CCN(CC3)C(=O)C3=CC4=C(O3)C=CC=C4)CC2)C=C1OC	7.356547
#	275	COC1=C2CCCC(CCCN3CCN(CC3)C3=C4C=CC=CC4=CC=C3)C2=CC=C1	6.452225
#	284	FC1=CC=CC=C1N1CCN(CCCCN2C(=O)OC3=CC=CC=C23)CC1	8.554396
#	287	O=C1OC2=C(C=CC=C2)N1CCCCN1CCN(CC1)C1CCCCC1	8.738
#	294	CC(=O)C1=CC=C2N(CCCCN3CCN(CC3)C3=CC=C(F)C=C3)C(=S)OC2=C1	8.725842
#	317	COC1=CC2=C(CN(CCCCC3=CN(C4=C3C=CC=C4)C3=CC=C(F)C=C3)C2)C=C1OC	7.269218
#	322	CC(COC1CC2CCC(C1)N2C)C1=CC=C(Br)C=C1	7.163043
#	325	[H]C1(CC2CCC(C1)N2CC1=CC=CC=C1)OC(=O)NC1=CC(C)=CC=C1OC	8.495
#	327	O=C1SC2=C(C=CC=C2)N1CCCCCCN1CCN(CC1)C1CCCCC1	8.827
#	33	COC1=CC2=C(NC=C2C(=O)CN2CCC(CC3=CC=CC=C3)CC2)C=C1	8
#	333	COC1=CC2=C(CN(CC3CCN(CC3)C(=O)C3=CC4=C(N3)C=CC=C4)CC2)C=C1OC	7.055517
#	348	CC(=O)C1=CC=C2N(CCCCN3CCN(CC3)C3=CC=C(F)C=C3)C(=O)OC2=C1	8.161781
#	353	COC1=C(OC)C=C(CNC2C3C4CC5C6CC(C3C46)C25)C=C1	7.602
#	360	CN(CC1=CC2=C(OCO2)C=C1)C1C2C3CC4C5CC(C2C35)C14	7.208
#	368	CN(CCCN1C(=O)OC2=CC3=C(C=C12)C(CC3=O)C1=CC=C(Cl)C=C1)CC1=CC=CC=C1	7.30103
#	374	COC1=CC=C(C=C1)[C@@H]1CC[C@@H](CC1)N1CCN(CC1)C1=NC=CC=C1	7.195179
#	378	OC12C3C4C5C3C(C3C5CC4C13)N2CC1=CC=NC=C1	6.289037
#	380	COC1=CC=CC2=C1CCC[C@@H]2NC(=O)CN1CCC2=CC(OC)=C(OC)C=C2C1	5.754241
#	391	CC1(C)C2CC[C@]1(C)CN(C2)C1CCCCC1	8.959
#	40	COC1=CC=C(CN2C=C(N=N2)C2=CC=C(C=C2)C(=O)NCCCCN2CCC3=CC(OC)=C(OC)C=C3C2)C=C1	7.50307
#	403	CN(CCCC1=CN(C2=C1C=CC=C2)C1=CC=C(F)C=C1)CC1=CC=C(C)C=C1C	7.329
#	412	FCCOCCOC1=CC=C(C=C1)N1CCN(CCCCC2=CN(C3=C2C=CC=C3)C2=CC=C(F)C=C2)CC1	6.339
#	416	CC1(C)C2CC[C@]1(C)CN(CCC1CCCC1)C2	9.155
#	420	CC1=CC=CC(NC(=O)OC2CC3CCCC(C2)N3CC2=CC=CC=C2)=C1C	8.309804
#	422	CC(=O)C1=CC=C2N(CCCCN3CCN(CC3)C3CCCCC3)C=CC2=C1	8.636
#	431	CCN(CCCCC1=CN(C2=C1C=CC=C2)C1=CC=C(F)C=C1)C1CCC2=C1C=CC(OC)=C2OC	7.559091
#	437	C(NC1C2C3CC4C5CC(C2C35)C14)C1CCCCC1	8.721246
#	441	CN(CCN(C)CCN1CCCC1)CCN(C)CCC1=CC(Cl)=C(Cl)C=C1	7.270835
#	451	C(CC1=CC=CC=C1)N1CCN(CC1)C1=CC=CC=N1	6.769551
#	454	COC1=C(OC)C2=C(C(NCCCCC3=CN(C4=CC=C(F)C=C4)C5=C3C=CC=C5)CC2)C=C1	6.939302
#	459	CN1CC2(CCN(CCCC(=O)C3=CC=C(F)C=C3)CC2)C2=CC=CC=C12	7.216096
#	478	CN(CCCN1CCCC1)CCN(C)CC1=CC(Cl)=C(Cl)C=C1	7.738
#	487	CCCCC1=CC=C(NC(=O)OC2CC3CCCC(C2)N3CC2=CC=CC=C2)C=C1	7.578396
#	496	CN1CCC2(CC1/C(=C/C1=CC=CC(Cl)=C1)C(=O)C2)C1=CC(O)=CC=C1	7.462181
#	511	COC1=CC=CC2=C1OCCC2CCCN1CCN(CC1)C1CCCCC1	8.012334
#	516	O=C1OC2=C(C=CC=C2)N1CCN1CCN(CC1)C1CCCCC1	8.2652
#	522	OC(CN1C2CCC1CC(C2)C1=CC=CC=C1)C1=CC=CC=C1	7.485452
#	524	O=C(C1=CC=C(C=C1NCCN2CC3=C(CC2)C=C(C(OC)=C3)OC)N4N=C(C5=C4CC(C)(CC5=O)C)C)N	6.086
#	532	CC(C1(CCN(CC1)[C@H]2[C@@H](C[C@@H](CC2)OCC3=CC=C(C=C3)F)O)C4=CC=CC=C4)=O	6.987163
#	66	COC1=C(OC)C=C2CN(CCCCNC(=O)C3=C(OC)C=CC(=C3)N3C=C(COCCOCCF)N=N3)CCC2=C1	7.180456
#	73	CN1CC[C@]2(C[C@@H]1/C(=C/C1=CC=CC(Cl)=C1)C(=O)C2)C1=CC(O)=CC=C1	7.65
#	74	COC1=C(OC)C=C2CN(CCCN3CCC4=CC(OC)=C(OC)C=C4C3=O)CCC2=C1	7.696804
#	77	CN1CC[C@]2(C[C@@H]1/C(=C/C1=CC=C(Cl)C=C1)C(=O)C2)C1=CC(O)=CC=C1	8.19382
#	80	BrC1=CC=C2OC(=O)N(CCCCN3CCN(CC3)C3CCCCC3)C2=C1	10.27572
#	86	COC1=CC=C(/C=C2/[C@H]3C[C@](CCN3C)(CC2=O)C2=CC(O)=CC=C2)C=C1	7.665546
*	1	COC1=C(OC)C=C2CN(CCCCN3C4=CC=CC=C4C4=C3C=CC=C4)CCC2=C1	10.39794
*	17	COC1=CC(CN2C=C(N=N2)C(=O)NCCCCN2CCC3=C(C2)C=C(OC)C(OC)=C3)=CC=C1	7.971
*	20	COC1=CC=C(CN2C=C(N=N2)C(=O)NCCN2CCC3=CC(OC)=C(OC)C=C3C2)C=C1	7.030584
*	29	FC1=CC=C(C=C1)C1(CC2CCC1C2)NCCCN1CCCCC1	7.79588
*	37	COC1=CC=C(Br)C=C1C(=O)NCCN1CCC2=CC(OC)=C(OC)C=C2C1	7.907
*	46	COC1=CC2=C(CN(CCCCNC(=O)C3=CN(CC4=CC=CC(O)=C4)N=N3)CC2)C=C1OC	7.647817
*	64	COC1=C(NC(=O)OC2CC3CCCC(C2)N3CCCCN)C=C(C)C=C1	7.888066
*	67	COC1=CC=CC(CN2C=C(N=N2)C2=CC=C(C=C2)C(=O)NCCCCN2CCC3=CC(OC)=C(OC)C=C3C2)=C1	7.179142
*	75	COC1=CC2=C(CN(CCCCNC(=O)C3=CC4=C(O3)C=CC(I)=C4)CC2)C=C1OC	8.208
*	79	COC1=C(OC)C=C2CN(CCCCNC(=O)C3=CC=C(C=C3)C3=CN(CCOCCF)N=N3)CCC2=C1	6.863
*	82	COC1=C2C(=O)N(CCCN3CCC4=CC(OC)=C(OC)C=C4C3)CCC2=CC=C1	7.787812
*	94	FC1=CC=C(C=C1)N1C(=O)N(CCCCN2CCN(CC2)C2=CC=C(F)C=C2)C2=CC=CC=C12	8.777284
*	106	CC(=O)C1=CC=C2N(CCCCN3CCN(CC3)C3=C(C=C(F)C=C3)[N+]([O-])=O)C(=O)OC2=C1	8.200659
*	130	COC1=C(NC(=O)OC2CC3CCCC(C2)N3CCCC2=CC=CC=C2)C=C(C)C=C1	8.69897
*	143	COC1=CC=CC(CN2C=C(N=N2)C2=CC=CC(=C2)C(=O)NCCCCN2CCC3=CC(OC)=C(OC)C=C3C2)=C1	7.180456
*	161	CN1C(=O)N(CCCCN2CCN(CC2)C2CCCCC2)C2=CC=CC=C12	8.590067
*	163	OC(CN1C2CCC1CC(C2)C1=CC=CC=C1)C1=C(Cl)C=CC=C1Cl	7.799423
*	168	COC1=CC=C(CN2C3C4C5C6C4C2(O)C2C6CC5C32)C=C1OC	7.899629
*	178	COC1=CC=C(CNCCCCC2=CN(C3=C2C=CC=C3)C2=CC=C(F)C=C2)C=C1OC	6.772
*	181	COC1=CC=CC2=C1CCC[C@@H]2NC(=O)CN1CCN(CC1)C1CCCCC1	8.228
*	187	CN(CC1=CC=CC=C1)C1C2C3CC4C5CC(C2C35)C14	7.30103
*	198	CN(CC1=CC(F)=CC=C1)C1C2C3CC4C5CC(C2C35)C14	6.931814
*	215	CC1(C)C2CC[C@]1(C)CN(CCC1CCCCCC1)C2	9.60206
*	221	COC1=CC(Br)=CC(C(=O)NCCCCN2CCN(CC2)C2=C(Cl)C(Cl)=CC=C2)=C1OC	7.124939
*	227	COC1=CC(OC)=C(NC(=O)OC2CC3CCCC(C2)N3CC2=CC=CC=C2)C=C1Cl	7.640165
*	239	FCCOC1=CC=C(C=C1)N1CCN(CCCCC2=CN(C3=C2C=CC=C3)C2=CC=C(F)C=C2)CC1	7.167
*	251	COC1=C(OC)C=C2CN(CCCNC(=O)CC3=CC=C(C=C3)C(=O)C3=CC=C(C=C3)[N+]([O-])=O)CCC2=C1	5.920096
*	258	CN(CCCCC1=CN(C2=C1C=CC=C2)C1=CC=C(F)C=C1)CC1=CC=CC(Cl)=C1	7.59176
*	261	[H][C@@]12CS[C@H](CCCCC(=O)NCCCCCC(=O)NCCCCCCN3C4CCCC3CC(C4)OC(=O)NC3=C(OC)C=CC(C)=C3)[C@]1([H])NC(=O)N2	5.869345
*	267	C(CC1=CC=CC=N1)N1C2CCC1CC2	6.286509
*	273	ClC1=CC=CC=C1[C@@H]1CC[C@@H](CC1)N1CCN(CC1)C1=NC=CC=C1	6.823909
*	283	ClC1=CC=CC=C1[C@H]1CC[C@@H](CC1)N1CCN(CC1)C1=NC=CC=C1	7.536107
*	293	S=C1OC2=C(C=CC=C2)N1CCCCN1CCN(CC1)C1CCCCC1	9.114
*	308	CC(C(=O)OC1CC2CCC(C1)N2C)C1=CC(Cl)=C(Cl)C=C1	6.509
*	321	CC(SC1=CC=CC=C1)C(=O)OC1CC2CCC(C1)N2C	6.498941
*	323	CC1(C)C2CC[C@]1(C)CN(CCCC1CCCCC1)C2	9.251812
*	330	COC1=C(NC(=O)OC2CC3CCCC(C2)N3CCCCCCNCC2=CC=C(Br)C=C2)C=C(C)C=C1	8.003
*	334	O=C1CCC2=CC=CN=C2N1CCCN1CCN(CC1)C1CCCCC1	7.793174
*	341	COC1=C(NC(=O)OC2CC3CCCC(C2)N3CC2=CC=CC=C2)C=C(C=C1)[N+]([O-])=O	7.777284
*	349	C1C[C@@H](CC[C@H]1N1CCN(CC1)C1=NC=CC=C1)C1=CC=CC=C1	7.57
*	357	COC1=C2C=CC=CC2=C(Br)C=C1C1=CC=C(CN2CC3=CC=CC=C3C2)N1	7.060481
*	364	OC1=CC2=C(NC=C2C(=O)CN2CCC(O)(CC2)C2=CC=C(Br)C=C2)C=C1	7.244125
*	370	O[C@@]12[C@H]3[C@@H]4[C@@H]5[C@H]3[C@@H]([C@H]3[C@@H]5C[C@@H]4[C@@H]13)N2CC1=CC=CC(I)=C1	7.268
*	377	CCCC1=CC=C2N(CCN3CCN(CC3)C3CCCCC3)C(=O)SC2=C1	8.314258
*	387	CC(CCCN1C(=O)SC2=CC=CC=C12)N1CCN(CC1)C1CCCCC1	8.501689
*	397	CC(OC1=CC(Cl)=C(Cl)C=C1)C(=O)OC1CC2CCC(C1)N2C	6.49485
*	406	CC(OC1=CC=C(Cl)C=C1)C(=O)OC1CC2CCC(C1)N2C	6.397723
*	413	COC1=CC(CNCCCCC2=CN(C3=C2C=CC=C3)C2=CC=C(F)C=C2)=CC=C1	6.644
*	418	OC12C3C4C5C3C(C3C5CC4C13)N2CCCC1=CC=CC(F)=C1	8.200659
*	421	S=C1SC2=C(C=CC=C2)N1CCCCN1CCN(CC1)C1CCCCC1	9.259637
*	423	CCC(COC1CC2CCC(C1)N2C)OC1=CC=C(Cl)C=C1	7.006564
*	433	COC1=C2CCCC(C(=O)NCCCN3CCN(CC3)C3CCCCC3)C2=CC=C1	7.783
*	438	O=C1SC2=C(C=CC=C2)N1CCN1CCN(CC1)C1CCCCC1	8.648
*	442	O=C1SC2=C(C=CC=C2)N1CCCCCN1CCN(CC1)C1CCCCC1	8.61261
*	452	[H]C1(CC2CCC(C1)N2CC1=CC=CC=C1)OC(=O)NC1=CC=C(Cl)C(Cl)=C1	7.548214
*	455	CCCC1=CC=C2N(CCN3CCN(CC3)C3CCCCC3)C(=O)OC2=C1	8.395774
*	477	COC1=CC=CC=C1[C@@H]1CC[C@@H](CC1)N1CCN(CC1)C1=NC=CC=C1	6.616185
*	480	O=C1OC2=CC=CC=C2N1CCCCN1CCN(CC1)C1=CC=CC=C1	7.942
*	490	O=C1OC2=C(C=CC=C2)N1CCCCCCN1CCN(CC1)C1CCCCC1	8.514279
*	497	CC1=CC=C(C=C1)C(=O)C1=CC=C(CCCN(CCCN2CCN(CC2)C2CCCCC2)CC#C)C=C1	7.7602
*	502	O=C(C1=CC=C(F)C=C1)C2CCN([C@@H]3CC(C=CC=C4OCCF)=C4C[C@H]3O)CC2	5.145998
*	515	O=C(C1=CC=C(F)C=C1)C2CCN([C@H]3CC(C(OCCF)=CC=C4)=C4C[C@@H]3O)CC2	5.108
*	518	COC1=CC(CCN2CCN(CCCC3=CC=CC=C3)CC2)=CC=C1OCC1=CC=CC=C1	7.784362
*	523	C(C1CCCCC1)N1C2CCC1CC2	7.744727
*	536	COC1=CC=CC(=C1)[C@H]1CC[C@@H](CC1)N1CCN(CC1)C1=NC=CC=C1	7.66

### 2.4 QSAR Hybrid model split 1 validation

The endpoints of the FDA-approved drugs were determined in order to additionally validate the model. The whole set composed of 1428 drugs was refined in order to remove quaternary ammonium salts, and compounds with too long SMILES (not elaborated by CORAL), and compounds containing atoms not enumerated in the model (Al, Fe, Gd, etc.). Overall, the whole set was reduced to 1376 compounds and these were evaluated with hybrid model resulting from split 1. Over 1376 compounds, 925 have been defined as outliers by the model since they fall outside the domain of applicability. Table 5 reports the SMILES and predicted σ_2_ p*K*_i_ for these FDA approved drugs evaluated with the hybrid model split 1.

**Table 5:** List of SMILES and predicted p*K*_i_ of the FDA-approved drugs.

	Calc. σ_2_ p*K*_i_
FC1=CC=C(C=C1)C(N1CCN(C/C=C/C2=CC=CC=C2)CC1)C1=CC=C(F)C=C1	9.1441
CCC1=NN(CCCN2CCN(CC2)C2=CC(Cl)=CC=C2)C(=O)N1CCOC1=CC=CC=C1	8.6407
FC1=CC=C(C=C1)C(CCCN1CCC2(CC1)N(CNC2=O)C1=CC=CC=C1)C1=CC=C(F)C=C1	8.6198
CCC(=O)N(C1CCN(CCC2=CC=CC=C2)CC1)C1=CC=CC=C1	8.6104
OC(CCN1CCCCC1)(C1CCCCC1)C1=CC=CC=C1	8.5665
FC1=CC=C(C=C1)C(CCCN1CCC(CC1)N1C(=O)NC2=CC=CC=C12)C1=CC=C(F)C=C1	8.5215
OC(CCN1CCCCC1)(C1CCCC1)C1=CC=CC=C1	8.5152
COC1=CC=C(C=C1)C(=O)NC1=CC=CC=C1CCC1CCCCN1C	8.4124
ClC1=CC2=C(C=C1)N(C1CCN(CCCN3C(=O)NC4=CC=CC=C34)CC1)C(=O)N2	8.322
OC(CCN1CCCC1)(C1CCCCC1)C1=CC=CC=C1	8.2572
CC(C)N(CC[C@H](C1=CC=CC=C1)C1=C(O)C=CC(C)=C1)C(C)C	8.2484
C(C(C1CCCCC1)C1CCCCC1)C1CCCCN1	8.1848
CN(C/C=C/C#CC(C)(C)C)CC1=CC=CC2=CC=CC=C12	8.0982
OC(CCCN1CCCCC1)(C1=CC=CC=C1)C1=CC=CC=C1	8.0673
CC(C)C1CC[C@@H](CC1)C(=O)N[C@H](CC1=CC=CC=C1)C(O)=O	8.0495
O=C(CCCC1=CC=CC=C1)OCC(COC(=O)CCCC1=CC=CC=C1)OC(=O)CCCC1=CC=CC=C1	8.0233
CN(CC=CC1=CC=CC=C1)CC1=CC=CC2=CC=CC=C12	7.9437
CCN(CCCC1=CC=CC=C1)CCCC1=CC=CC=C1	7.9234
FC1=CC=C(C=C1)C(=O)CCCN1CCC(=CC1)N1C(=O)NC2=CC=CC=C12	7.9139
CC(C)N(CCC(C(N)=O)(C1=CC=CC=C1)C1=CC=CC=N1)C(C)C	7.8974
C(C=CC1=CC=CC=C1)N1CCN(CC1)C(C1=CC=CC=C1)C1=CC=CC=C1	7.8956
OC(=O)C1(CCN(CCC(C#N)(C2=CC=CC=C2)C2=CC=CC=C2)CC1)C1=CC=CC=C1	7.845
CN(CC1=CC=C(C=C1)C(C)(C)C)CC1=CC=CC2=CC=CC=C12	7.8042
CN(C)C1=C(C)N(C)N(C1=O)C1=CC=CC=C1	7.7999
CC/C(=C(/C1=CC=CC=C1)C1=CC=C(OCCN(C)C)C=C1)C1=CC=CC=C1	7.7675
CCN(CC)CCCN(C1CC2=CC=CC=C2C1)C1=CC=CC=C1	7.7595
CC(COC1=CC=CC=C1)N(CCCl)CC1=CC=CC=C1	7.737
COC1=CC=C(C=C1)C(Cl)=C(C1=CC=C(OC)C=C1)C1=CC=C(OC)C=C1	7.7352
CC(C)(C(O)=O)C1=CC=C(C=C1)C(O)CCCN1CCC(CC1)C(O)(C1=CC=CC=C1)C1=CC=CC=C1	7.7321
OC(CCN1CCCCC1)(C1CC2CC1C=C2)C1=CC=CC=C1	7.7316
NCCCC[C@H](N[C@@H](CCC1=CC=CC=C1)C(O)=O)C(=O)N1CCC[C@H]1 C(O)=O	7.7236
CC(C)(C)C1=CC=C(C=C1)C(O)CCCN1CCC(CC1)C(O)(C1=CC=CC=C1)C1=CC=CC=C1	7.7088
CC1=CC(CN2CCN(CC2)C(C2=CC=CC=C2)C2=CC=C(Cl)C=C2)=CC=C1	7.6641
COC1=C(C=C(C=C1)C1=CC2=C(C=C1)C=C(C=C2)C(O)=O)C12CC3CC(CC(C3)C1)C2	7.6161
OC(=O)C(CC(=O)N1CC2CCCCC2C1)CC1=CC=CC=C1	7.6118
FC1=CC=C(C=C1)[C@@H]1CCNC[C@H]1COC1=CC2=C(OCO2)C=C1	7.573
OC1(CCN(CCCC(=O)C2=CC=C(F)C=C2)CC1)C1=CC=C(Cl)C=C1	7.5648
CCCCC1C(=O)N(N(C1=O)C1=CC=CC=C1)C1=CC=CC=C1	7.5128
CN(C(=S)OC1=CC2=CC=CC=C2C=C1)C1=CC=CC(C)=C1	7.4966
CC(C)CC(N(C)C)C1(CCC1)C1=CC=C(Cl)C=C1	7.4951
CC1(C)CCC(C)(C)C2=C1C=CC(NC(=O)C1=CC=C(C=C1)C(O)=O)=C2	7.4322
C1=CN(C=N1)C(C1=CC=CC=C1)C1=CC=C(C=C1)C1=CC=CC=C1	7.4322
NC1=C2CCCCC2=NC2=CC=CC=C12	7.4304
CC(C)(C)C1=CC=C(CN2CCN(CC2)C(C2=CC=CC=C2)C2=CC=C(Cl)C=C2)C=C1	7.422
CN1CCC(CC1)OC(C1=CC=CC=C1)C1=CC=CC=C1	7.4197
CN1CCN(CC1)C(C1=CC=CC=C1)C1=CC=CC=C1	7.4185
CN1CCN(CC1)C1=NC2=CC=CC=C2OC2=C1C=C(Cl)C=C2	7.3641
CN1CCN2C(C1)C1=CC=CC=C1CC1=CC=CC=C21	7.3411
COC1=C(OC)C=C2C(=O)C(CC3CCN(CC4=CC=CC=C4)CC3)CC2=C1	7.3381
CN(C)CCOC(C1=CC=CC=C1)C1=CC=C(Br)C=C1	7.3083
CC1=CC=CC=C1C(=O)NC1=CC(C)=C(C=C1)C(=O)N1CCC[C@@H](O)C2=C1C=CC(Cl)=C2	7.3058
CN(C)CCOC1=CC=C(C=C1)C(=C(/CCCl)C1=CC=CC=C1)/C1=CC=CC=C1	7.2901
CCN(CC)CCOC1=CC=C(C=C1)C(=C(Cl)C1=CC=CC=C1)C1=CC=CC=C1	7.2583
CN1CCC2=C(C1)C(C1=CC=CC=C21)C1=CC=CC=C1	7.2133
NC(=O)C([C@@H]1CCN(CCC2=CC3=C(OCC3)C=C2)C1)(C1=CC=CC=C1)C1=CC=CC=C1	7.2095
[O-][N+](=O)C1=CC=C(O1)C=NN1CCOC1=O	7.1872
C[C@@H](CC1=CC=CC=C1)N(C)CC1=CC=CC=C1	7.1849
CN(C)CCOC(C)(C1=CC=CC=C1)C1=CC=CC=N1	7.1638
CN1N(C(=O)C=C1C)C1=CC=CC=C1	7.1634
CN1CCN2C(C1)C1=CC=CC=C1CC1=C2N=CC=C1	7.1368
CN1CCCN=C1COC(=O)C(O)(C1CCCCC1)C1=CC=CC=C1	7.1047
[O-][N+](=O)C1=CC=C(C=C1)C1=CC=C(O1)C=NN1CC(=O)NC1=O	7.1001
CCC1=C(CC)C=C2CC(CC2=C1)NC[C@H](O)C1=C2C=CC(=O)NC2=C(O)C=C1	7.0973
COC1=CC=C(CCN2CCC(CC2)NC2=NC3=CC=CC=C3N2CC2=CC=C(F)C=C2)C=C1	7.0965
CN(C)CCOC(C1=CC=CC=C1)C1=CC=CC=C1C	7.0904
CN(C)CCOC(C1=CC=CC=C1)C1=CC=CC=C1	7.0898
ClC1=CC=C(COC(CN2C=CN=C2)C2=C(Cl)C=C(Cl)C=C2)C=C1	7.086
COC1=CC=C(C=C1)N1N=C(C(N)=O)C2=C1C(=O)N(CC2)C1=CC=C(C=C1)N1CCCCC1=O	7.0795
FC1=CC=C(C=C1)N1C=C(C2CCN(CCN3CCNC3=O)CC2)C2=C1C=CC(Cl)=C2	7.0782
CN1CCC(CC1)=C1C2=CC=CC=C2C=CC2=CC=CC=C12	7.0691
COC(=O)[C@@H]([C@H]1CCCCN1)C1=CC=CC=C1	7.0488
C(N(CC1=CC=CC=C1)C1=CC=CC=C1)C1=NCCN1	7.0166
CNC1(C)C2CCC(C2)C1(C)C	7.0002
CCCNCC(O)COC1=CC=CC=C1C(=O)CCC1=CC=CC=C1	6.9943
CC(CNC1CCCCC1)OC(=O)C1=CC=CC=C1	6.9751
O[C@@H](CNCCCCC1=CC=C(O)C=C1)C1=CC(O)=C(O)C=C1	6.96
C[C@@H](CC1=CC(O)=C(O)C=C1)[C@H](C)CC1=CC(O)=C(O)C=C1	6.9306
CCC(=C(CC)C1=CC=C(O)C=C1)C1=CC=C(O)C=C1	6.9195
C[C@](N)(CC1=CC=C(O)C=C1)C(O)=O	6.9117
C[C@H](NCCC1=CC=C(O)C=C1)[C@H](O)C1=CC=C(O)C=C1	6.9076
C[C@](N)(CC1=CC(O)=C(O)C=C1)C(O)=O	6.8954
CC1=CC(=O)N(O)C(=C1)C1CCCCC1	6.8813
CCN(CC)CCOC(=O)C1(CCCCC1)C1CCCCC1	6.8762
COC(=O)C(C1CCCCN1)C1=CC=CC=C1	6.8738
CN1CCN(CC2=CC=C(NC(=O)C3=CC(C#CC4=CN=C5C=CC=NN45)=C(C)C=C3)C=C2C(F)(F)F)CC1	6.8607
ClC1=CC(Cl)=C(COC(CN2C=CN=C2)C2=C(Cl)C=C(Cl)C=C2)C=C1	6.8594
C[C@@H](NCCCC1=CC(=CC=C1)C(F)(F)F)C1=CC=CC2=CC=CC=C12	6.8412
NNCCC1=CC=CC=C1	6.8077
CC(N)C12CC3CC(CC(C3)C1)C2	6.7976
CN[C@H]1CC[C@@H](C2=CC(Cl)=C(Cl)C=C2)C2=CC=CC=C12	6.7952
ClC1=CC2=C(OC3=CC=CC=C3N=C2N2CCNCC2)C=C1	6.7952
CC1(C)C(C=C(Cl)Cl)C1C(=O)OCC1=CC(OC2=CC=CC=C2)=CC=C1	6.7806
CC1=CC=C(C=C1)N(CC1=NCCN1)C1=CC(O)=CC=C1	6.7556
COC1=CC=C(C=C1)C(CN(C)C)C1(O)CCCCC1	6.7523
CN(C)CCC=C1C2=CC=CC=C2CCC2=CC=CC=C12	6.7503
OC1=CC=C(C=C1)C1=C(C(=O)C2=CC=C(OCCN3CCCCC3)C=C2)C2=C(S1)C=C(O)C=C2	6.7472
CCC1(C)OC(=O)N(C)C1=O	6.7467
CN1CCC(CC1)=C1C2=CC=CC=C2CCC2=C1N=CC=C2	6.7408
CC=C(C(=CC)C1=CC=C(O)C=C1)C1=CC=C(O)C=C1	6.738
C(C1=NCCN1)C1=CC=CC2=CC=CC=C12	6.7366
COC1=CC2=C(C=C1)C=C(C=C2)[C@H](C)C(O)=O	6.7337
ClC1=CC=CC(=C1)N1CCN(CCCN2N=C3C=CC=CN3C2=O)CC1	6.7229
CCC(C1=CC=CC=C1)C1=C(O)C2=C(OC1=O)C=CC=C2	6.7189
ClC1=CC2=C(C=C1)N(CC1CC1)C(=O)CN=C2C1=CC=CC=C1	6.7128
CCOC(=O)C1(CCN(CCC(C#N)(C2=CC=CC=C2)C2=CC=CC=C2)CC1)C1=CC=CC=C1	6.7032
CCCCN1CCCC[C@H]1 C(=O)NC1=C(C)C=CC=C1C	6.6889
CN(C)CCCN1C2=CC=CC=C2CCC2=CC=CC=C12	6.6606
CC1=CC2=C(C=C1C(=C)C1=CC=C(C=C1)C(O)=O)C(C)(C)CCC2(C)C	6.6603
O=C1CC2(CCCC2)CC(=O)N1CCCCN1CCN(CC1)C1=NC=CC=N1	6.6561
ClC1=CC2=C(C=C1)C(=C1CCNCC1)C1=C(CC2)C=CC=N1	6.6391
OC(=O)C1=CC=C(C=C1)N1N=C(N=C1C1=CC=CC=C1O)C1=CC=CC=C1O	6.6384
CCCCOC1=CC=C(C=C1)C(=O)CCN1CCCCC1	6.6313
CCCN1CCCC[C@H]1 C(=O)NC1=C(C)C=CC=C1C	6.6184
CC(CCC1=CC=C(O)C=C1)NCCC1=CC(O)=C(O)C=C1	6.6156
CCC1(C(=O)NC(=O)N(C)C1=O)C1=CC=CC=C1	6.6102
CN(C)CCOC(C1=CC=C(Cl)C=C1)C1=CC=CC=N1	6.6073
CCC1(NC(=O)N(C)C1=O)C1=CC=CC=C1	6.6012
CC1=CC(=NN=C1NCCN1CCOCC1)C1=CC=CC=C1	6.5948
CN(C)CCC=C1C2=CC=CC=C2C=CC2=CC=CC=C12	6.5864
CN(C)CCCN1C2=CC=CC=C2CCC2=C1C=C(Cl)C=C2	6.5832
OC(=O)C1=CC=C(NC(=O)[C@H](CC2=CC=C(O)C=C2)NC(=O)C2=CC=CC=C2)C=C1	6.5741
CN1CCCCC1C(=O)NC1=C(C)C=CC=C1C	6.5716
CN(C)CCC(C1=CC=CC=C1)C1=CC=CC=N1	6.5683
CN(C)CCN(CC1=CC=CC=C1)C1=CC=CC=N1	6.5662
NC[C@H]1CC[C@@H](CC1)C(O)=O	6.556
CN(C)CCN(CC1=CC=C(Cl)C=C1)C1=CC=CC=N1	6.548
OC(=O)C1=CC(=CC=C1O)/N=N/C1=CC=C(O)C(=C1)C(O)=O	6.5425
CN1C2=C(C=C(Cl)C=C2)N(C2=CC=CC=C2)C(=O)CC1=O	6.5409
CN(C)CC[C@@H](C1=CC=C(Br)C=C1)C1=CC=CC=N1	6.5328
[H]C(CCN(C)C)=C1C2=CC=CC=C2COC2=CC=CC=C12	6.5251
COC1=C(OC)C=C(CCNCC(O)COC2=CC=CC(C)=C2)C=C1	6.5119
CN(C)CC(C1=CC=C(O)C=C1)C1(O)CCCCC1	6.5098
CCC#CC(C)C1(CC=C)C(=O)NC(=O)N(C)C1=O	6.5086
CCCCN1CCCCC1C(=O)NC1=C(C)C=CC=C1C	6.4996
CC(C(O)=O)C1=CC2=C(C=C1)C1=C(N2)C=CC(Cl)=C1	6.496
OC(=O)C1=CC=CC=C1OC(=O)C1=CC=CC=C1O	6.4944
C#CCN[C@@H]1CCC2=CC=CC=C12	6.4937
CC1=C(OC2=C(C=CC=C2C(=O)OCCN2CCCCC2)C1=O)C1=CC=CC=C1	6.491
CCN1CC(CCN2CCOCC2)C(C1=O)(C1=CC=CC=C1)C1=CC=CC=C1	6.4576
CC1=NN(C(=O)/C1=N/NC1=C(O)C(=CC=C1)C1=CC=CC(=C1)C(O)=O)C1=CC=C(C)C(C)=C1	6.457
CC1=CC=C(C=C1)C(=C/CN1CCCC1)/C1=CC=CC=N1	6.4355
CCOC(=O)C1(CCN(CCC2=CC=C(N)C=C2)CC1)C1=CC=CC=C1	6.4344
CCOC(=O)N1CCC(CC1)=C1C2=C(CCC3=C1N=CC=C3)C=C(Cl)C=C2	6.4249
CN(CC#C)CC1=CC=CC=C1	6.4243
CC(C)COCC(CN(CC1=CC=CC=C1)C1=CC=CC=C1)N1CCCC1	6.4243
CCC1=C(C(N)=NC(N)=N1)C1=CC=C(Cl)C=C1	6.424
FC(F)(F)COC1=CC(C(=O)NCC2CCCCN2)=C(OCC(F)(F)F)C=C1	6.4101
NC(=O)N1C2=CC=CC=C2C=CC2=CC=CC=C12	6.4094
CN1CCN(CC1)C1=NC2=CC(Cl)=CC=C2NC2=CC=CC=C12	6.3913
OC1=CC=C(OCC2=CC=CC=C2)C=C1	6.3895
CC(C)CCC[C@@H](C)CCC[C@@H](C)CCC/C(C)=C/CC1=C(C)C(=O)C2=C(C=CC=C2)C1=O	6.3882
CCN1CCC[C@H]1CNC(=O)C1=C(OC)C=CC(Br)=C1OC	6.3872
BrCCC(=O)N1CCN(CC1)C(=O)CCBr	6.3859
CC(C)NCC(O)COC1=CC=CC2=CC=CC=C12	6.3853
CC(N)COC1=C(C)C=CC=C1C	6.3676
CN(C)CCOC(=O)C(C1=CC=CC=C1)C1(O)CCCC1	6.3626
CN(C)CCC(C1=CC=C(Cl)C=C1)C1=CC=CC=N1	6.3602
CC(CC1=CC=CC=C1)N(C)CC#C	6.3534
CC(C)(C)NCC(O)C1=CC(Cl)=C(N)C(Cl)=C1	6.3499
CC(C)(N)CC1=CC=CC=C1	6.348
CC(C1=C(CCN(C)C)CC2=CC=CC=C12)C1=CC=CC=N1	6.3475
CC(N)CC1=CC=CC=C1	6.3469
O=C(OCC1=CC=CC=C1)C1=CC=CC=C1	6.3448
CN1CCCC1C1=CN=CC=C1	6.3363
CN[C@@H]1CCC2=C(C1)C1=C(N2)C=CC(=C1)C(N)=O	6.3275
COC1=C(OC)C=C2C3CC(=O)C(CC(C)C)CN3CCC2=C1	6.3214
CCN(C)C(=O)OC1=CC=CC(=C1)[C@H](C)N(C)C	6.3202
COC1=CC=CC=C1OCCNCC(O)COC1=CC=CC2=C1C1=CC=CC=C1N2	6.3105
CN(C)CCC1=CNC2=CC=C(C[C@H]3COC(=O)N3)C=C12	6.3046
NC12CC3CC(CC(C3)C1)C2	6.2988
CC[C@@H](N1CCCC1=O)C(N)=O	6.2978
CC(C)[C@@H]1CC[C@@H](C)C[C@H]1O	6.2947
COC1=CC=CC=C1OCC(O)CN1CCN(CC(=O)NC2=C(C)C=CC=C2C)CC1	6.2856
CC(C)NCC(O)COC1=CC=C(CCOCC2CC2)C=C1	6.2783
COC1=C(O)C=C(C=C1)[C@@H]1CC(=O)C2=C(O)C=C(O)C=C2O1	6.2783
CNCCCC1C2=CC=CC=C2C=CC2=CC=CC=C12	6.275
CNCCC(OC1=CC=C(C=C1)C(F)(F)F)C1=CC=CC=C1	6.2707
CN1N=C(C(=O)NC2CC3CCCC(C2)N3C)C2=CC=CC=C12	6.2701
CNCCC=C1C2=CC=CC=C2CCC2=CC=CC=C12	6.2659
CC1C(OCCN1C)C1=CC=CC=C1	6.2651
NCCC1=CC(O)=C(O)C=C1	6.2639
CC[C@H]1[C@@H](CC2=CN=CN2C)COC1=O	6.251
CC1NCCOC1C1=CC=CC=C1	6.249
CC[C@H](C)[C@H](N)C(O)=O	6.2448
CCC1(CCC(=O)NC1=O)C1=CC=CC=C1	6.225
CN(C)C1=NC(=NC(=N1)N(C)C)N(C)C	6.2209
CN(C)CCC(C1=CC=C(Br)C=C1)C1=CC=CC=N1	6.2091
CC(=O)OCC(=O)NCCCOC1=CC=CC(CN2CCCCC2)=C1	6.2082
CCNC1C2CCC(C2)C1C1=CC=CC=C1	6.2068
CCOC(=O)N1C=CN(C)C1=S	6.1954
CNC(C)(C)CC1=CC=CC=C1	6.1803
CCC1(CC)C(=O)NC(=O)N(C)C1=O	6.1802
CNCCCN1C2=CC=CC=C2CCC2=CC=CC=C12	6.1763
CCN(C(=O)C=CC)C1=CC=CC=C1C	6.1721
CC1=C(C=NO1)C(=O)NC1=CC=C(C=C1)C(F)(F)F	6.1691
CCN(CC)CCNC(=O)C1=CC(Cl)=C(N)C=C1OC	6.1583
CCC1(CCC(=O)NC1=O)C1=CC=C(N)C=C1	6.1484
CC1=C(C)C(NC2=CC=CC=C2C(O)=O)=CC=C1	6.1474
[H]C(Cl)=CC(O)(CC)C#C	6.1414
CCOC(=O)[C@H](CCC1=CC=CC=C1)N[C@@H](C)C(=O)N1CCC[C@H]1 C(O)=O	6.1296
CC(CCC1=CC=CC=C1)NCC(O)C1=CC(C(N)=O)=C(O)C=C1	6.1275
CCOC(=O)NC1=C(N)C=C(NCC2=CC=C(F)C=C2)C=C1	6.1062
CCC1(CC)C(=O)NCC(C)C1=O	6.0791
C[C@H](N)[C@H](O)C1=CC(O)=C(O)C=C1	6.074
CCN(CC)CCOC(=O)C1=C(Cl)C=C(N)C=C1	6.0661
CC(C(O)=O)C1=CC(F)=C(C=C1)C1=CC=CC=C1	6.0655
CC(CC1=CC=C(O)C=C1)NCC(O)C1=CC(O)=CC(O)=C1	6.0602
COC1=CC(=CC(OC)=C1OC)C(=O)NCC1=CC=C(OCCN(C)C)C=C1	6.0573
C(C1=NCCN1)C1=CC=CC=C1	6.0359
COC1=C(OC)C=C2C(N)=NC(=NC2=C1)N1CCN(CC1)C(=O)C1COC2=CC=CC=C2O1	6.0338
COC1=CC=C(CN(CCN(C)C)C2=NC=CC=C2)C=C1	6.0332
CC1=CC(=CC(C)=C1CC1=NCCN1)C(C)(C)C	6.0224
CN1C(=O)CC(C)(C1=O)C1=CC=CC=C1	6.008
ClC1=CC=C(C=C1)C(=O)NCCN1CCOCC1	6.0071
CC[C@H]([C@@H](C)CN(C)C)C1=CC(O)=CC=C1	5.9943
CN/C(NCC1=CC=CC=C1)=N/C	5.9826
COC1=CC(O)=C(C=C1)C(=O)C1=CC=CC=C1	5.9764
ClC1=CC2=C(OC(=O)N2)C=C1	5.9722
OC(=O)C1=C(O)C=CC(=C1)C1=C(F)C=C(F)C=C1	5.9689
COC1=C(C/C=C(/C)CCC(=O)OCCN2CCOCC2)C(O)=C2C(=O)OCC2=C1C	5.9683
NC(=O)N1C2=CC=CC=C2CC(=O)C2=CC=CC=C12	5.9628
CC12CC3CC(C)(C1)CC(N)(C3)C2	5.9506
CC1=CC(OCCCC(C)(C)C(O)=O)=C(C)C=C1	5.9395
CCCOC1=C(N)C=C(C=C1)C(=O)OCCN(CC)CC	5.9382
CN(C)CCC1=CNC2=C1C=C(CN1C=NC=N1)C=C2	5.9287
COC1=CC(NC(C)CCCN)=C2N=CC=CC2=C1	5.9265
CNC(C)CC1CCCCC1	5.9229
COC1=CC=C(C=C1)C1C(=O)C2=CC=CC=C2C1=O	5.9048
CC(C(O)=O)C1=CC(OC2=CC=CC=C2)=CC=C1	5.9022
C[C@@H](CC1=CC=CC=C1)NC(=O)[C@@H](N)CCCCN	5.8946
CCC1=C(C)NC2=C1C(=O)C(CN1CCOCC1)CC2	5.8943
N[C@@H](CC1=CNC2=CC=CC=C12)C(O)=O	5.892
COC1=C2OC(=O)C=CC2=CC2=C1OC=C2	5.8901
CN(C(=O)C(Cl)Cl)C1=CC=C(OC(=O)C2=CC=CO2)C=C1	5.8799
OC1=C(Cl)C=C(Cl)C2=C1N=CC=C2	5.8705
N[C@H]([C@@H](O)C1=CC(O)=C(O)C=C1)C(O)=O	5.862
[O-][N+](=O)C1=CC2=C(NC(=O)CN=C2C2=CC=CC=C2Cl)C=C1	5.8484
CCOC(=O)C1(CCN(C)CC1)C1=CC=CC=C1	5.838
OC1=C([C@H]2CC[C@@H](CC2)C2=CC=C(Cl)C=C2)C(=O)C2=CC=CC=C2C1=O	5.822
ClC1=CC2=C(NC(=O)CN=C2C2=CC=CC=C2Cl)C=C1	5.8184
CN1C(=O)CC(C1=O)C1=CC=CC=C1	5.8124
OC(=O)C1=CC=CC=C1O	5.8083
NC(=N)N1CCC2=CC=CC=C2C1	5.7825
[O-][N+](=O)C1=CC=C(O1)/C=N/N1CC(=O)NC1=O	5.7824
CC(C)(C)NCC(O)COC1=CC=CC2=C1CCC(=O)N2	5.7793
ClC1=CC(Cl)=C(CO/N=C(/CN2C=CN=C2)C2=C(Cl)C=C(Cl)C=C2)C=C1	5.7711
CC1=NC2=C(CCN(C(=O)C3=CC=C(NC(=O)C4=CC=CC=C4C4=CC=CC=C4)C=C3)C3=CC=CC=C23)N1	5.7639
CC(C)[C@H](N)C(O)=O	5.7636
CC1CC(CC(C)(C)C1)OC(=O)C(O)C1=CC=CC=C1	5.76
CCN(CC)CCOC(=O)C1=CC=C(N)C=C1	5.756
CCCCOC1=C(N)C=CC(=C1)C(=O)OCCN(CC)CC	5.7534
FC1=CC=C(C=C1)C(N1CCN(CC1)C1=NC(NCC=C)=NC(NCC=C)=N1)C1=CC=C(F)C=C1	5.7486
CN1C(=O)NC(=O)C(C)(C1=O)C1=CCCCC1	5.7352
OC1=CC=CC=C1	5.7276
CCC(=O)NCC[C@@H]1CCC2=C1C1=C(OCC1)C=C2	5.7259
CN(C)CCCN1C2=CC=CC=C2SC2=C1C=C(C=C2)C(C)=O	5.7251
CN1C=CNC1=S	5.7185
OC1N=C(C2=CC=CC=C2)C2=C(NC1=O)C=CC(Cl)=C2	5.7127
N[C@@H](CC1=CC=CC=C1)C(O)=O	5.6852
C[C@@H](O)[C@H](N)C(O)=O	5.6773
OC1=C(CC2=C(O)C3=C(OC2=O)C=CC=C3)C(=O)OC2=C1C=CC=C2	5.6753
N[C@@H](CC1=CC=C(O)C=C1)C(O)=O	5.6666
O=C(C1CCCCC1)N1CC2N(CCC3=CC=CC=C23)C(=O)C1	5.6603
CCOC(=O)C1=CC=C(N)C=C1	5.655
CN[C@@H](C)[C@@H](O)C1=CC=CC=C1	5.6468
CCCCC1(CC)C(=O)NC(=O)NC1=O	5.6428
CC1=CC(OCC2CNC(=O)O2)=CC(C)=C1	5.641
C[C@@H](N)[C@@H](O)C1=CC=CC=C1	5.6385
NC1=NC(N)=C2N=C(C(N)=NC2=N1)C1=CC=CC=C1	5.6326
CN[C@@H](C)[C@H](O)C1=CC=CC=C1	5.6255
COC1=CC(C(O)C(C)N)=C(OC)C=C1	5.6252
CC(C(O)=O)C1=CC(=CC=C1)C(=O)C1=CC=CC=C1	5.6186
CCN(CC)CCCC(C)NC1=C2C=C(OC)C=CC2=NC2=C1C=CC(Cl)=C2	5.6149
C[C@H](N)[C@H](O)C1=CC(O)=CC=C1	5.6108
OC1N=C(C2=CC=CC=C2Cl)C2=C(NC1=O)C=CC(Cl)=C2	5.6067
NC1=NC(N)=C(C=C1)/N=N/C1=CC=CC=C1	5.6043
CCOC(=O)C(C)(C)OC1=CC=C(Cl)C=C1	5.5982
N[C@@H](CC1=CC(O)=C(O)C=C1)C(O)=O	5.5951
NC1=CC(=NC(N)=[N+]1[O-])N1CCCCC1	5.5938
CC(C)NCC(O)COC1=CC=CC2=C1C=CN2	5.5904
CCOC1=C(C=CC(CC(=O)N[C@@H](CC(C)C)C2=CC=CC=C2N2CCCCC2)=C1)C(O)=O	5.5881
NC1=CC2=NC3=C(C=CC(N)=C3)C=C2C=C1	5.5872
CC(C)C1=C(OCC2=NCCN2)C=C(C)C=C1	5.5839
OC(=O)P(O)(O)=O	5.581
CCC1(C(=O)NC(=O)NC1=O)C1=CCCCCC1	5.578
CN1C2=C(C=C(Cl)C=C2)C(=NC(O)C1=O)C1=CC=CC=C1	5.5742
NCCC1=CC=NN1	5.5635
CC1=CNN=C1	5.5628
[O-][N+](=O)C1=C(C=CC(=C1)C(F)(F)F)C(=O)C1C(=O)CCCC1=O	5.5615
CC(C)NCC(O)COC1=CC=C(COCCOC(C)C)C=C1	5.5568
NC1=CC(C(O)=O)=C(O)C=C1	5.5191
NC(=O)C1=CC=CC=C1O	5.5173
CNC[C@H](O)C1=CC(O)=C(O)C=C1	5.5172
CCC1(CCC(C)C)C(=O)NC(=O)NC1=O	5.5159
CN1CCCC(CC1)N1N=C(CC2=CC=C(Cl)C=C2)C2=CC=CC=C2C1=O	5.5105
CC(N)C(=O)NC1=C(C)C=CC=C1C	5.5082
CNCCCC12CCC(C3=CC=CC=C13)C1=CC=CC=C21	5.5018
O=C1C(C(=O)C2=CC=CC=C12)C1=CC=CC=C1	5.4923
C[C@H](C1=CNC=N1)C1=C(C)C(C)=CC=C1	5.4682
NC1=CC(Cl)=C(NC2=NCCN2)C(Cl)=C1	5.4667
CC1=CC(=O)C2=CC=CC=C2C1=O	5.4471
NC1=CC(O)=C(C=C1)C(O)=O	5.4335
CN1CCN(CC(=O)N2C3=CC=CC=C3C(=O)NC3=C2N=CC=C3)CC1	5.4294
CCN1C(=O)NC(C1=O)C1=CC=CC=C1	5.4294
CN1C(=O)OC(C)(C)C1=O	5.4232
COC1=C(OC)C=C2C(N)=NC(=NC2=C1)N(C)CCCNC(=O)C1CCCO1	5.4112
CCOC(=O)C1=CN=CN1[C@H](C)C1=CC=CC=C1	5.4103
CC1=CC(OCC(O)CNC(C)(C)C)=C(Cl)C=C1	5.4088
CC(C)C1=CC=CC(C(C)C)=C1O	5.3957
OC(=O)C1CCN2C1=CC=C2C(=O)C1=CC=CC=C1	5.3933
NCCC[C@H](N)C(O)=O	5.3882
N[C@@H](CC1=CNC2=C1C=C(O)C=C2)C(O)=O	5.3762
NCCCC[C@H](N)C(O)=O	5.3748
ClC1=CC=CC(Cl)=C1NC1=NCCN1	5.3673
CCN(CC)C(=O)N1CCN(C)CC1	5.3635
CCC1(C(=O)NC(=O)NC1=O)C1=CC=CC=C1	5.3352
CN1C=NC2=C1C(=O)N(C)C(=O)N2C	5.3313
O[Bi]1OC(=O)C2=CC=CC=C2O1	5.3308
CN(C)C(=O)OC1N=C(C2=CC=CC=C2)C2=C(C=CC(Cl)=C2)N(C)C1=O	5.3306
C(N1CCCNCCNCCCNCC1)C1=CC=C(CN2CCCNCCNCCCNCC2)C=C1	5.3094
CC(C)(C)NCC(O)C1=CC(O)=CC(O)=C1	5.3086
O[C@@H]1CNC(C1)C(O)=O	5.3025
CC1=C2NC(=O)C3=C(N=CC=C3)N(C3CC3)C2=NC=C1	5.2855
CC(C)NCC(O)C1=CC(O)=C(O)C=C1	5.283
CCC1(C(=O)NCNC1=O)C1=CC=CC=C1	5.2768
CNCCC1=CC=CC=N1	5.273
CN1C=CC(=O)C(O)=C1C	5.2728
OC(=O)[C@@H]1CCCN1	5.264
BrC1=C(NC2=NCCN2)C=CC2=NC=CN=C12	5.2565
CN1C=NC2=C1C(=O)NC(=O)N2C	5.2435
CN(C)C(=O)CC1=C(N=C2C=CC(C)=CN12)C1=CC=C(C)C=C1	5.2346
N[C@@H](CC1=CC=C(C=C1)N(CCCl)CCCl)C(O)=O	5.2212
OC(=O)C1N=C(C2=CC=CC=C2)C2=C(NC1=O)C=CC(Cl)=C2	5.2194
CCC1(C)CC(=O)NC1=O	5.2159
N[C@@H]1CONC1=O	5.2032
CC(O)C(O)C1CNC2=C(N1)C(=O)N=C(N)N2	5.1999
CC(C)(C(=O)C1=CN=CC=C1)C1=CN=CC=C1	5.1996
CCN1C=C(C(O)=O)C(=O)C2=CC(F)=C(C=C12)N1CCN(C)CC1	5.1989
NC(=N)C1=CC=C(OCCCCCOC2=CC=C(C=C2)C(N)=N)C=C1	5.1966
CNC[C@H](O)C1=CC(O)=CC=C1	5.1892
CC1=CC(=C(O)C(C)=C1CC1=NCCN1)C(C)(C)C	5.1871
CC(C)CC1=CC=C(C=C1)C(C)C(O)=O	5.1703
CC(=O)OC1=CC=CC=C1C(O)=O	5.1669
CC(NC(C)(C)C)C(=O)C1=CC(Cl)=CC=C1	5.1655
[H][C@]12CC3=C(C(O)=C(O)C=C3)C3=CC=CC(CCN1C)=C23	5.1542
COC1=C(OC)C=C2C(N)=NC(=NC2=C1)N1CCN(CC1)C(=O)C1CCCO1	5.1478
COC1=CC(C(O)CNC(=O)CN)=C(OC)C=C1	5.1441
FC1=CNC(=O)NC1=O	5.1318
CCCN(CCC)CCC1=C2CC(=O)NC2=CC=C1	5.1113
CCN(CC)CCCC(C)NC1=C2C=CC(Cl)=CC2=NC=C1	5.1096
NC1=CC=CC2=C1CN(C1CCC(=O)NC1=O)C2=O	5.1068
CCN(CC)CCNC1=C2C(=O)C3=CC=CC=C3SC2=C(C)C=C1	5.1055
OC(C1CCCCN1)C1=CC(=NC2=C1C=CC=C2C(F)(F)F)C(F)(F)F	5.0966
CCN(CC)C(=O)C1CN2CCC3=CC(OC)=C(OC)C=C3C2CC1OC(C)=O	5.0825
CCN(CC)CC1=C(O)C=CC(NC2=C3C=CC(Cl)=CC3=NC=C2)=C1	5.0701
[O-][N+](=O)C1=CC2=C(NC(=O)CN=C2C2=CC=CC=C2)C=C1	5.0685
CC(C)NCC(O)COC1=CC=CC=C1CC=C	5.0593
C[C@H](N)C(O)=O	5.057
CC(C)NCC(O)C1=CC(O)=CC(O)=C1	5.0536
CN1C2=C(C=C(Cl)C=C2)C(=NCC1=O)C1=CC=CC=C1	5.0478
CCN(CC)CC(=O)NC1=C(C)C=CC=C1C	5.0421
CNC1=NC2=C(C=C(Cl)C=C2)C(C2=CC=CC=C2)=N(=O)C1	5.0419
ClCCNP1(=O)OCCCN1CCCl	5.0259
CCC(C)C1(CC)C(=O)NC(=O)NC1=O	5.0059
CCN1C=C(C(O)=O)C(=O)C2=CC(F)=C(C=C12)N1CCNCC1	5.0044
CC1=CC2=CC3=C(OC(=O)C=C3C)C(C)=C2O1	4.9947
NC1=CC=CC2=C1C(=O)N(C1CCC(=O)NC1=O)C2=O	4.9875
CN(C)N=NC1=C(N=CN1)C(N)=O	4.9857
CC(C)NCC(O)COC1=CC=CC=C1OCC=C	4.9808
CC(C)(C)C1=CC(=C(O)C=C1NC(=O)C1=CNC2=CC=CC=C2C1=O)C(C)(C)C	4.9796
COC1=C(OC)C=C2C(N)=NC(=NC2=C1)N1CCN(CC1)C(=O)C1=CC=CO1	4.9672
FC(F)(F)C(Cl)Br	4.9575
N[C@@H](CN1C=CC(=O)C(O)=C1)C(O)=O	4.9447
BrC1=CC2=C(NC(=O)CN=C2C2=CC=CC=N2)C=C1	4.9444
COC1=C(OC)C=C(CC2=NC=CC3=CC(OC)=C(OC)C=C23)C=C1	4.9297
CCN(CC)CCNC(=O)C1=CC=C(N)C=C1	4.9257
FC(F)(F)C1=CC(=CC=C1)N1CCN(CCOC(=O)C2=CC=CC=C2NC2=C3C=CC(=CC3=NC=C2)C(F)(F)F)CC1	4.9228
OC(=O)C1=CN=CC=C1	4.9182
CCCC1=NC2=C(C=C(C=C2C)C2=NC3=CC=CC=C3N2C)N1CC1=CC=C(C=C1)C1=CC=CC=C1C(O)=O	4.9152
CC(C)CC1(CC=C)C(=O)NC(=O)NC1=O	4.8842
COC1=CC(CC2=CN=C(N)N=C2N)=CC(OC)=C1OC	4.8739
CC[C@H](NC(C)C)[C@H](O)C1=C2C=CC(=O)NC2=C(O)C=C1	4.8724
NC1=CC(=CNC1=O)C1=CC=NC=C1	4.8713
CCN1C=C(C(O)=O)C(=O)C2=CC(F)=C(N=C12)N1CCNCC1	4.8712
CC(C)/N=C(/N)N=C(N)NC1=CC=C(Cl)C=C1	4.8623
CCN1N=C(C(O)=O)C(=O)C2=CC3=C(OCO3)C=C12	4.8524
NC(=O)C1=CN(CC2=C(F)C=CC=C2F)N=N1	4.8467
NC1=CC=NC=C1	4.8453
COC(F)(F)C(Cl)Cl	4.8088
CCN[C@@H](C)CC1=CC=CC(=C1)C(F)(F)F	4.795
CN1C2=C(C=C(Cl)C=C2)C(=NCC1=O)C1=CC=CC=C1F	4.793
CCOCCN1C(=NC2=CC=CC=C12)N1CCCN(C)CC1	4.7658
CC(C)C(=O)C1=C2C=CC=CN2N=C1C(C)C	4.7495
ClCCN(CCCl)C1=CNC(=O)NC1=O	4.734
CCC(NC(C)C)C(O)C1=CC(O)=C(O)C=C1	4.7239
CC(=O)NC1=CC=C(O)C=C1	4.7184
ClC1=CC=C(NC(=N)NC(=N)NCCCCCCNC(=N)NC(=N)NC2=CC=C(Cl)C=C2)C=C1	4.7115
COC1=CC(NCC2=C(C)C3=C(C=C2)N=C(N)N=C3N)=CC(OC)=C1OC	4.7007
CN(CCCl)CCCl	4.6918
FC(F)(F)CN1C2=C(C=C(Cl)C=C2)C(=NCC1=O)C1=CC=CC=C1	4.6896
CCCC1=CC(=O)NC(=S)N1	4.6845
CN1C2=C(C3=CC=CC=C13)C(=O)N(CC1=C(C)NC=N1)CC2	4.6758
COC(=O)CCC1=CC=C(OCC(O)CNC(C)C)C=C1	4.6736
CCN1C=C(C(O)=O)C(=O)C2=C1C=C(C=C2)C1=CC=NC=C1	4.6709
NC1=C2NC=NC2=NC=N1	4.659
CCCN1C2=C(NC=N2)C(=O)NC1=O	4.6231
CC(=O)N(O)CCCCCNC(=O)CCC(=O)N(O)CCCCCNC(=O)CCC(=O)N(O)CCCCCN	4.6191
CCCNC(C)C(=O)NC1=CC=CC=C1C	4.5857
CN1N=NC2=C(N=CN2C1=O)C(N)=O	4.5784
OC(=O)C1=C(NC2=CC=CC(=C2)C(F)(F)F)N=CC=C1	4.5744
CC(C)C1=CC2=C(OC3=NC(N)=C(C=C3C2=O)C(O)=O)C=C1	4.5664
CNC(C)CCC=C(C)C	4.5579
CC(C)C1(CC=C)C(=O)NC(=O)NC1=O	4.5566
CCCC(C)C1(CC)C(=O)NC(=O)NC1=O	4.5539
CCN(CC)C(C)C(=O)C1=CC=CC=C1	4.5459
CCC(C)C1(CC=C)C(=O)NC(=O)NC1=O	4.5302
O=C1N=CN=C2NNC=C12	4.5171
CC(C)C[C@H](N)C(O)=O	4.4648
CCCC(CCC)C(O)=O	4.4645
FC(F)OC(F)(F)C(F)Cl	4.4242
C1CNCCN1	4.4238
CCCCCCOC(=O)N=C(N)C1=CC=C(NCC2=NC3=C(C=CC(=C3)C(=O)N(CCC(=O)OCC)C3=CC=CC=N3)N2C)C=C1	4.3803
CCOC(=O)C1=C2CN(C)C(=O)C3=C(C=CC(F)=C3)N2C=N1	4.3715
CCN(CC)CCNC(=O)C1=C(C)NC(/C=C2/C(=O)NC3=C2C=C(F)C=C3)=C1C	4.3589
CC(C)N=C1C=C2N(C3=CC=C(Cl)C=C3)C3=C(C=CC=C3)N=C2C=C1NC1=CC=C(Cl)C=C1	4.3349
CC(=O)C(O)=O	4.3271
CC(C)CN1C=NC2=C1C1=CC=CC=C1N=C2N	4.2934
ClCCN(CCCl)P1(=O)NCCCO1	4.2917
CCCCOC1=NC2=CC=CC=C2C(=C1)C(=O)NCCN(CC)CC	4.279
CCCC(C)C1(CC=C)C(=O)NC(=O)NC1=O	4.2435
CCCC1=C2N(CC)C(=CC(=O)C2=CC2=C1OC(=CC2=O)C(O)=O)C(O)=O	4.2409
CN1C2=C(NC=N2)C(=O)N(C)C1=O	4.2262
CC(C)C[C@H](NC(=O)[C@H](CC1=CC=CC=C1)NC(=O)C1=CN=CC=N1)B(O)O	4.2023
N[C@@H](CC1=CN=CN1)C(O)=O	4.1816
NC1=C(F)C=NC(=O)N1	4.1626
CCN1C=C(C(O)=O)C(=O)C2=C1N=C(C)C=C2	4.1483
CC(C)[C@H](N)C(=O)OCCOCN1C=NC2=C1NC(N)=NC2=O	4.1155
CN1CCN(CC2=CC=C(C=C2)C(=O)NC2=CC(NC3=NC=CC(=N3)C3=CN=CC=C3)=C(C)C=C2)CC1	4.0658
CN1C2=C(C3=CC=CC=C13)C(=O)C(CN1C=CN=C1C)CC2	4.058
CCOC1=C(OCC)C=C(/C=C2/NCCC3=CC(OCC)=C(OCC)C=C23)C=C1	4.0101
CCCCNC1=CC=C(C=C1)C(=O)OCCOCCOCCOCCOCCOCCOCCOCCOCCOC	3.9748
C[C@@H](CN1CC(=O)NC(=O)C1)N1CC(=O)NC(=O)C1	3.9464
NCCCNCCCCNCCCN	3.8504
CC1=NC=C2CN=C(C3=CC=CC=C3F)C3=C(C=CC(Cl)=C3)N12	3.8476
CCN1C=C(C(O)=O)C(=O)C2=CC(F)=C(N3CCNC(C)C3)C(F)=C12	3.8329
CCCC(=O)NC1=CC(C(C)=O)=C(OCC(O)CNC(C)C)C=C1	3.595
NC(=O)C1=NC=CN=C1	3.4337
FC(F)OC(F)C(F)(F)F	3.389
CNCC(O)C1=CC(OC(=O)C(C)(C)C)=C(OC(=O)C(C)(C)C)C=C1	3.3212
CC(C)(C)C(=O)OCOP(=O)(COCCN1C=NC2=C(N)N=CN=C12)OCOC(=O)C(C)(C)C	2.9868
CCC1NC(=O)C(C(O)C(C)C/C=C/C)N(C)C(=O)C(C(C)C)N(C)C(=O)C(CC(C)C)N(C)C(=O)C(CC(C)C)N(C)C(=O)C(C)NC(=O)C(C)NC(=O)C(CC(C)C)N(C)C(=O)C(NC(=O)C(CC(C)C)N(C)C(=O)CN(C)C1=O)C(C)C	1.5436
